# Motor cortex directly excites the substantia nigra pars reticulata, the basal ganglia output nucleus

**DOI:** 10.1038/s41467-026-74569-w

**Published:** 2026-06-23

**Authors:** William Scott Thompson, Patryk Wekwejt, Sten Grillner, Gilad Silberberg

**Affiliations:** Department of Neuroscience, Karolinska Institutet, Stockholm, Sweden

**Keywords:** Basal ganglia, Motor cortex

## Abstract

Inhibitory neurons of the substantia nigra pars reticulata (SNr) serve as a primary output through which the basal ganglia regulate behavior. Using a virally targeted optogenetic approach, combined with whole cell patch-clamp recordings of SNr neurons, we show that, in mice, projection neurons of both primary and secondary motor cortices (M1 and M2) form monosynaptic excitatory connections onto different subpopulations of GABAergic SNr neurons. Furthermore, photostimulation of these cortical axon terminals markedly increases SNr neuron firing rate. To investigate the spatial organization of cortical input to the SNr, we employed a transsynaptic viral-labelling approach to identify SNr neurons receiving monosynaptic input from either M1 or M2. We found a topographical organization of the M1 and M2 projections in SNr. Chemogenetic inhibition of M1- and M2-targeted SNr neurons induced opposing changes in spontaneous behavior. These findings reveal functional pathways by which the motor cortex can directly modulate basal ganglia output to downstream targets.

## Abstract (web summary)

The cortex shapes the output of the basal ganglia via multiple polysynaptic synaptic pathways. Here, a direct pathway from two cortical motor regions to the output nucleus of the basal ganglia, substantia nigra pars reticulata, is described.

## Introduction

Successful motor output requires coordination between the plethora of brain structures that make up the motor system. The basal ganglia are a set of subcortical nuclei that integrate multiple streams of information, to select and reinforce context appropriate motor programs^1^. Located in the ventral midbrain, the substantia nigra pars reticulata (SNr) is the largest output nucleus of the rodent basal ganglia and serves as an important link between the integrative processing of the basal ganglia and motor circuits in the forebrain, midbrain, and brainstem^2–4^.

GABAergic SNr neurons are intrinsically active and directly regulate behavioral output through tonic inhibition of their downstream targets in the midbrain and brainstem^5–10^. They also transmit efference copy information back upstream, via collaterals to thalamic nuclei^4,11^. Rather than forming a broad global output, distinct groups of GABAergic SNr neurons project to unique sets of targets^4^. While most SNr neurons express parvalbumin (PV), target-defined subpopulations are spatially clustered, and both genetically and electrophysiologically distinguishable^4,12^. SNr projection patterns reflect a modular organization of the basal ganglia as a whole, whereby information flow is organized into parallel modules for specialized control of individual motor components^11,13–15^. The rate and pattern of SNr neuron firing in vivo are shaped by synaptic input. Canonically, the primary inputs to GABAergic SNr neurons originate from upstream basal ganglia nuclei, namely: the striatum, globus pallidus pars externa (GPe), and the subthalamic nucleus (STN) via the so-called direct, indirect, and hyperdirect pathways^16–18^. The SNr also receives input from structures outside the basal ganglia^19,20^. Compared to the intrinsic connectivity within the basal ganglia, little is known about the role that these external inputs play in modulating SNr activity.

Conventionally partitioned into primary and secondary subdivisions (herein abbreviated as M1 and M2, respectively), the rodent motor cortex is essential for motor learning and behavioral flexibility, as well as the coordination of skilled motor output^21–27^. Projection neurons of both M1 and M2 emit complex descending axons: individual neurons innervate several target structures through the basal ganglia, thalamus, midbrain, brainstem, and spinal cord^27–32^. In rodents, anatomical evidence suggests that terminals of cortical axons are also found within the substantia nigra^15,27–29,32–34^. Retrograde transsynaptic tracing has identified both M1 and M2 as sources of input to the dopaminergic substantia nigra population^35^. However, whether the motor cortex forms functional synapses with the GABAergic population of SNr neurons is unknown.

In the present study, we sought to investigate the functional synaptic transmission from the motor cortex to the GABAergic neurons of the SNr. We employed a virally targeted optogenetic approach, whereby we could selectively activate cortical axons, and performed ex vivo whole cell recordings to assess postsynaptic responses in GABAergic SNr neurons. With the addition of pharmacological protocols, we establish the existence of a monosynaptic connection between glutamatergic neurons in the motor cortex and GABAergic SNr neurons. We characterize the synaptic properties of this connection and show that optogenetic activation of this pathway affects the firing rate of SNr neurons ex vivo. To further understand the spatial distribution of cortical-recipient SNr neurons we employed a transsynaptic viral labeling approach. Our findings show that M1 and M2 target different subpopulations of SNr neurons. Chemogenetic manipulations of the two subpopulations of cortical-recipient SNr neurons in vivo produced contrasting effects on spontaneous behavior. These results characterize functional cortical pathways from M1 and M2 that provide direct external regulation of basal ganglia output.

## Results

### Monosynaptic excitation of GABAergic SNr neurons by M1 projections

**Fig. 1:**
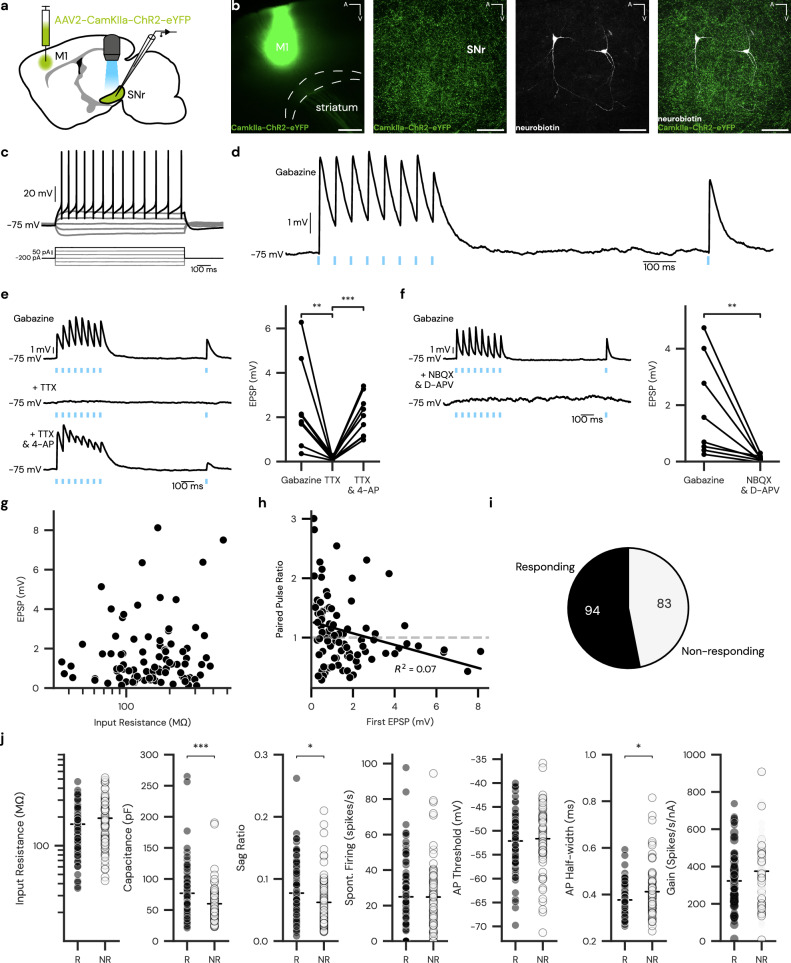
Photostimulation of M1 axon terminals evokes EPSPs in a subpopulation of GABAergic SNr neurons. **a** Experimental setup. Channelrhodopsin (ChR2) expression was driven in projection neurons of M1 via injection of a viral vector (AAV2-CamkIIa-ChR2-eYFP). SNr neurons were recorded in acute brain slices via whole-cell patch clamp, in current clamp configuration. **b** Representative image of injection site in M1, in a parasagittal brain slice, and confocal image of corresponding fluorescently labeled axon terminals within the SNr. Addition of neurobiotin to the intracellular solution allowed for post-hoc visualization of neuron morphology relative to fluorescently labeled M1 axon terminals (A: anterior, V: ventral). **c** GABAergic cell identity was assessed by characteristic responses to current pulses. **d** Excitatory postsynaptic potentials (EPSPs) were recorded in response to trains of photostimulation (2 ms, 1 mW; stimulation onset indicated by blue marks) during bath application of the GABA_A_ antagonist gabazine (10 µM). An injected hyperpolarizing current kept the baseline membrane potential at approximately −75 mV. **e** Left: Representative response to photostimulation in the presence of gabazine (upper), gabazine and the sodium channel blocker TTX (1 µM) (middle), and gabazine, TTX, and the voltage-gated potassium channel blocker 4-AP (100 μM) (lower). Right: summary of EPSP amplitudes recorded in the conditions represented in the left panel (*n* = 9 neurons; ∗∗ *p* < 0.01, ∗∗∗ *p* < 0.001; two-sided Wilcoxon signed-rank test). **f** Left: photostimulation in the presence of gabazine (upper), and gabazine combined with glutamate receptor antagonists NBQX (10 µM) and D-APV (50 µM) (lower). Right: summary of EPSP amplitudes recorded in the conditions represented in the left panel (*n* = 8 neurons; ∗∗ *p* < 0.01; two-tailed paired *t* test). **g** Scatterplot of EPSP amplitudes and neuron input resistance from the recorded population. **h** Scatterplot of first EPSP amplitude and paired pulse ratio. Solid black line indicates linear regression; dashed gray line indicates a paired pulse ratio of 1. **i** Proportion of recorded neurons that responded to photostimulation; numbering represents total counts. **j** Comparison of selected electrophysiological features between responding (R) and non-responding (NR) neurons (*n* = 94 neurons, *N* = 31 animals; ∗ *p* < 0.05, ∗∗∗ *p* < 0.001, two-sided Mann–Whitney U). Mean values represented by horizontal bars. Scale bars: **b** 500 µm (left), 100 µm (others).

To gain an experimental handle on motor cortex efferents, we injected AAV2-CamKIIa-ChR2-eYFP unilaterally in M1 and obtained whole cell patch clamp recordings from SNr neurons in acute brain slices (Fig. 1a, b). This approach offered expression of both the fluorescent reporter protein eYFP and the excitatory opsin Channelrhodopsin (ChR2) selectively in M1 projection neurons, allowing us to simultaneously visualize and photostimulate efferent axons while recording from SNr neurons in their vicinity (Fig. 1a). The cell-type identity of recorded neurons was confirmed as either dopaminergic or GABAergic based on characteristic electrophysiological signatures (Fig. 1c, Supplementary Fig. 1)^10^.

Excitatory postsynaptic potentials (EPSPs) were evoked by widefield photostimulation, delivered as 20 Hz trains of 2 ms long light pulses (Fig. 1d). Recordings were performed under continuous bath application of the GABA_A_ receptor antagonist gabazine (10 μM) to occlude concurrent synaptic activity stemming from either the striatum, GPe, or from neighboring SNr neurons. In a subset of experiments (*n* = 9 neurons), EPSPs were abolished by bath application of the sodium channel antagonist Tetrodotoxin (TTX, 1 μM), confirming that the observed potentials were synaptic in nature. EPSPs were, however, restored by subsequent bath application of the voltage-gated potassium channel antagonist 4-AP (100 μM), indicating that responses were monosynaptic in nature (Fig. 1e)^36^. In a further subset of experiments (*n* = 8 neurons), EPSPs were abolished by bath application of glutamatergic receptor antagonists NBQX (10 μM) and D-APV (50 μM), confirming the glutamatergic nature of the synapses (Fig. 1f).

From a holding potential of −75 mV, EPSPs presented with amplitudes ranging up to 8 mV (Fig. 1g). The recorded EPSP amplitudes were independent of the input resistance of the postsynaptic neurons (*p* = 0.11, r^2^ = 0.028; *n* = 94 neurons; *N* = 31 animals; Fig. 1g). This lack of correlation suggests that synaptic contacts are sparsely distributed across the dendritic arborization^37,38^. SNr neurons possess long, passive dendrites, thought to be important for the integration of competing signals (Supplementary Fig. 2)^39,40^.

Photostimulation with a series of pulses at 20 Hz allows the engagement of short-term synaptic plasticity, while remaining within the kinetic activation range of the opsin (ChR2). Short-term plasticity at corticofugal synapses is highly variable, even within the same postsynaptic cell type^41^. Similarly, when recording from SNr neurons, we observed both depressing and facilitating synaptic responses (Fig. 1h). A weak, inverse correlation was found between the measured paired pulse ratio and the amplitude of the first EPSP, with smaller amplitude synapses tending to exhibit facilitation (*p* = 0.008, *r*^2^ = 0.07, *n* = 94 neurons; *N* = 31 animals).

The majority (53%) of GABAergic SNr neurons from which we recorded responded to photostimulation of cortical efferents (*n* = 94 of 177 neurons; Fig. 1i). All neurons were subject to a battery of current injections to profile their intrinsic electrophysiological properties. Commonly reported sub- and suprathreshold electrophysiological properties were compared between responding (R) and non-responding neurons (NR). Responding neurons exhibited significantly higher membrane capacitance (R: 77.13 ± 4.21 pF, NR: 60.11 ± 3.14 pF; *p* = 0.0006), an increased sag ratio (R: 0.077 ± 0.005, NR: 0.063 ± 0.004; *p* = 0.028), and shorter action potential half-width (R: 0.377 ± 0.007 ms, NR: 0.412 ± 0.012 ms; *p* = 0.032) relative to non-responding neurons (*n* = 94 (R), *n* = 83 (NR); *N* = 31 animals; Fig. 1j). No significant differences were observed between the two groups in terms of input resistance, spontaneous firing rate and action potential threshold. Target-defined SNr subpopulations display distinct electrophysiological signatures^4^. Thus, the electrophysiological differences between responding and non-responding neurons may hint to a circuit motif whereby M1 selectively targets a specific set of SNr neurons.

Altogether, these results demonstrate that glutamatergic M1 projections form functional, monosynaptic connections with a population of GABAergic SNr neurons.

### Monosynaptic excitation of GABAergic SNr neurons by M2 projections

**Fig. 2:**
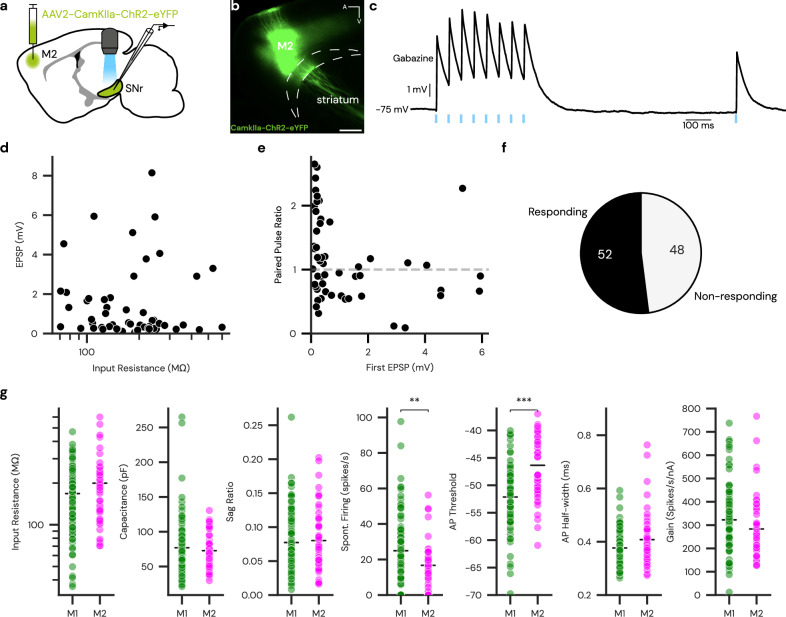
Photostimulation of M2 axon terminals evokes EPSPs in a subpopulation of GABAergic SNr neurons. **a** Experimental setup. ChR2 expression was driven in projection neurons of M2 via injection of a viral vector (AAV2-CamkIIa-ChR2-eYFP; A: anterior, V: ventral). SNr neurons were recorded in acute brain slices via whole-cell patch clamp, in current clamp configuration. **b** Representative widefield image of injection site in M2, in a parasagittal brain slice. **c** EPSPs were recorded in response to trains of photostimulation (2 ms, 1 mW; stimulation onset indicated by blue marks) during bath application of the GABA_A_ antagonist gabazine (10 µM). Hyperpolarizing current was injected to keep the baseline membrane potential at approximately −75 mV. **d** Scatterplot of EPSP amplitudes and neuron input resistance from the recorded population (*n* = 52 neurons, *N* = 20 animals). **e** Scatterplot of first EPSP amplitude and paired pulse ratio. Horizontal dashed gray line indicates a paired pulse ratio of 1. **f** Proportion of recorded neurons that responded to photostimulation; numbering represents total counts. 52% of neurons tested responded to M2 terminal photostimulation (*n* = 52 of 100 neurons; *N* = 11 animals; Fig. 3F). **g** Comparison of selected electrophysiological features between M1-responding (M1) and M2-responding (M2) neurons (*n* = 94, 52 neurons; *N* = 31, 20 animals ∗ *p* < 0.05, ∗∗ *p* < 0.01, ∗∗∗ *p* < 0.001, two-sided Mann–Whitney U). Mean values represented by horizontal bars. Scale bar: **b** 500 µm.

The secondary motor cortex (M2) also has extensive direct projections to subcortical structures^42^. To assess whether M2 neurons target the GABAergic population of the SNr, we injected AAV2-CamKIIa-ChR2-eYFP unilaterally in M2, and recorded from SNr neurons, as previously detailed (Fig. 2a, b). We recorded EPSPs in response to widefield photostimulation (Fig. 2c). As with M1-SNr transmission, EPSP amplitude was not correlated to the input resistance of the postsynaptic neuron (*p* = 0.95, *R*^2^ < 0.0001; *n* = 52 neurons; *N* = 11 animals; Fig. 2d). M2-SNr EPSPs demonstrated a mix of facilitation and depression, with no correlation between the amplitude of the first EPSP in the train and the recorded paired pulse ratio (*p* = 0.22, *R*^2^ = 0.028; *n* = 52 neurons; *N* = 11 animals; Fig. 2e). Most of the SNr neurons recorded responded to photostimulation of M2 axons (*n* = 52 of 100 neurons, Fig. 2f).

Projections from M1 and M2 maintain a topographic organization as they innervate target structures in the subcortical motor system^15,27,43^. As the intrinsic electrophysiological properties of SNr neurons also adhere to a topographical organization^4,44^, we reasoned that M1 and M2 may target different populations within the SNr. We compared M1-responding to M2-responding neurons along common electrophysiological properties (Fig. 2g). While no differences were observed in subthreshold properties, M1-responding cells displayed significantly higher spontaneous firing rate (M1: 24.7 ± 1.9 spikes/s, M2: 16.7 ± 2.6 spikes/s; *p* = 0.0029) and significantly lower action potential threshold (M1: −52.0 ± 0.6 mV, M2: −46.3 ± 0.9 mV; *p* < 0.0001; M1: *n* = 94 neurons, *N* = 31 animals; M2: *n* = 52 neurons, *N* = 11 animals; Fig. 2g). Together, these results demonstrate that M2 innervates a population of SNr neurons that electrophysiologically differ from the M1-recipient population.

### Pre- and postsynaptic selectivity of corticonigral projections

Whereas most recorded neurons displayed electrophysiological features characteristic of GABAergic SNr neurons, dopaminergic neurons within the SNr were occasionally encountered (Supplementary Fig. 1, 2). Of this dopaminergic population, only a minority responded to photostimulation of either M1- or M2-efferent axons (*n* = 3 of 39 neurons; Supplementary Fig. 1).

To assess whether other cortical areas project to the SNr, we employed the same experimental strategy, but with the virus targeted to the primary somatosensory cortex (S1; Supplementary Fig. 3). S1 projection neurons target the main input structure of the basal ganglia, the striatum, in a similar fashion to M1 projections^41^. However, S1 injections did not yield any labeled axons at the level of the SNr, and no recorded SNr neurons responded to photostimulation (*n* = 27 neurons, *N* = 4 animals). Thus, we did not find any evidence for direct synaptic transmission from primary somatosensory cortex to the SNr.

### Cortical input to SNr neurons is mediated by AMPA receptors

**Fig. 3:**
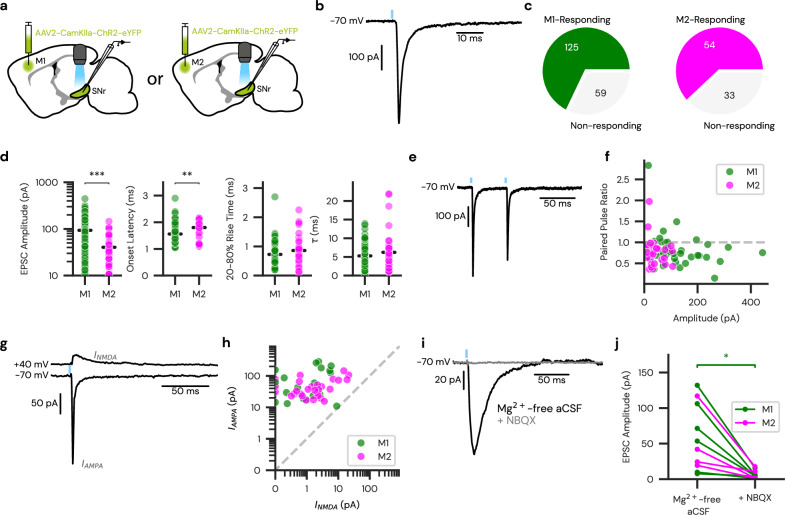
Characterization of optogenetically evoked EPSCs from M1/M2 axon terminals. **a** Experimental setup. ChR2 expression was driven in projection neurons of either M1 or M2 via injection of a viral vector (AAV2-CamkIIa-ChR2-eYFP). SNr neurons were recorded in acute brain slices via whole-cell patch clamp, in voltage clamp configuration in the presence of gabazine. **b** Representative excitatory postsynaptic current (EPSC) recorded in response to photostimulation (2 ms, 1 mW; stimulation onset indicated by blue mark). **c** Proportion of recorded neurons that responded to photostimulation in voltage clamp configuration; numbering represents total counts. **d** Comparison of absolute EPSC amplitude and kinetics (onset latency, 20–80% rise time and decay time constant) between M1-evoked and M2-evoked EPSCs: absolute EPSC amplitude, (M1: *n* = 91 neurons, *N* = 20 animals; M2: *n* = 41 neurons, *N* = 9 animals; ∗∗ *p* < 0.01; ∗∗∗ *p* < 0.001; two-sided Mann–Whitney U). Mean values indicated by horizontal bars. **e** In a subset of experiments, paired light pulses were delivered at 50 ms intervals (2 ms, 1 mW; stimulation onset indicated by blue mark). **f** Scatterplot of first EPSC amplitude and paired pulse ratio for neurons tested with paired pulse protocol (M1: *n* = 44 neurons,* N* = 11 animals; M2: *n* = 36 neurons, *N* = 8 animals). Horizontal dashed gray line indicates a paired pulse ratio of 1. **g** In a subset of experiments, EPSCs were recorded at both −70 and +40 mV to measure the relative contribution of AMPA and NMDA receptors. **h** Scatterplot of absolute AMPA and NMDA currents recorded (M1: *n* = 15 neurons, *N* = 5 animals; M2: *n* = 28 neurons, *N* = 7 animals). Dashed black line indicates I_NMDA_ = I_AMPA_. **i:** In a further subset of experiments, EPSCs were recorded in a Mg^2+^-free solution before (black trace), and after bath application of the AMPA receptor antagonist NBQX (gray trace). **j** Summary of EPSC amplitudes recorded in Mg^2+^-free solution and after bath application of NBQX (M1: *n* = 6 neurons, *N* = 1 animal; M2: *n* = 4 neurons, *N* = 2 animals; ∗ *p* < 0.05; two-sided Wilcoxon signed-rank test).

The kinetics and short-term dynamics of postsynaptic responses to M1 input vary considerably between different postsynaptic cell types within the basal ganglia^41,45^. For a thorough characterization of corticonigral synapses we recorded from SNr neurons in voltage clamp configuration while photostimulating either M1 or M2 axon terminals (Fig. 3a, b). Across all experiments in voltage clamp configuration, we detected excitatory postsynaptic currents (EPSCs) in 64% of neurons tested with M1 stimulation, and 62% of neurons tested with M2 stimulation (M1: *n* = 125 of 194 neurons; *N* = 26 animals; M2: *n* = 54 of 87 neurons, *N* = 11 animals; Fig. 3c). The increased proportion of respondent cells observed in voltage-clamp relative to current clamp configuration may reflect that cortical terminals may impinge upon the dendritic domain of SNr neurons. With a potassium-based intracellular solution, synaptic events arriving at distal dendrites may be less detectable due to dendritic filtering^46^. Our voltage-clamp intracellular solution was cesium-based, offering improved space clamping. EPSCs evoked by M1 axon stimulation were significantly higher in amplitude than those evoked by M2 axon stimulation (M1: 93.40 ± 8.67 pA; *n* = 91; *N* = 20; M2: 40.48 ± 4.91 pA; *n* = 41; *N* = 9 Fig. 2d). EPSCs consistently presented with short onset latencies (i.e. less than 3 ms—as expected for monosynaptic connections), although the onset latency of M1 synapses was slightly shorter than their M2 counterparts (M1: 1.56 ± 0.35 ms; *n* = 91; *N* = 20; M2: 1.79 ± 0.06 ms; *n* = 41; *N* = 9; Fig. 3d). No differences were observed between M1-efferent and M2-efferent EPSC rise and decay times (Fig. 3d). In a subset of experiments, we again assessed the short-term plasticity of corticonigral synapses by measuring the paired pulse ratio in response to two consecutive light pulses (Fig. 3e). For M1-evoked responses, we found a mean paired pulse ratio of 0.83 (*n* = 44), reflecting a majority of depressing synapses (*n* = 36/44). Similar results were obtained for M2-evoked responses: a mean paired pulse ratio of 0.79, reflecting 34 of 36 synapses that demonstrated short term depression. In comparing the paired pulse ratio to the amplitude of the first EPSC we found no significant correlation in either case (M1: *p* = 0.11, R^2^ = 0.06; M2: *p* = 0.15; R^2^ = 0.06; Fig. 3f). Differences between these results and those observed in current clamp recordings (Fig. 1h) may be attributed to the respective recording configuration and intracellular solutions^47^.

Postsynaptic responses to glutamatergic signaling are largely mediated by the expression of N-methyl-D-aspartate (NMDA) and a-amino-3-hydroxy-5-methyl-4-isoxazolepropionic (AMPA) receptors^48^. To assess the relative contributions of these two receptor types at the corticonigral synapse, we separated the AMPA receptor-mediated and NMDA receptor-mediated EPSC components by photostimulating while holding the neuron at either negative or positive potentials (Fig. 3g). We found a near complete domination of AMPA-mediated over NMDA-mediated current at both M1- and M2-efferent synapses (Fig. 3h). To further corroborate this result, we performed recordings in a Mg^2+^-free extracellular solution, thus removing the voltage-dependent Mg^2+^ gating of NMDA receptors^49,50^. Optically evoked EPSCs recorded in this Mg^2+^-free environment could be blocked by bath application of the AMPA receptor antagonist NBQX (Fig. 3i, j). These results demonstrate that corticonigral synaptic transmission is primarily AMPA receptor mediated.

Calcium-permeable AMPA (CP-AMPA) receptors are prevalent in fast-spiking neuron types across the brain^41,51,52^. This receptor class is electrophysiologically identifiable due to strong polyamine-dependent inward rectification^53^. To assess whether CP-AMPA receptors are present at corticonigral synapses, we obtained voltage clamp recordings of optically evoked EPSCs (as above) using a polyamine-containing intracellular solution, across different holding potentials (Supplementary Fig. 4). Both M1-SNr and M2-SNr synapses displayed inward rectification characteristic of CP-AMPA receptors, suggesting the possibility of calcium-dependent plasticity at corticonigral synapses.

### Photostimulation of motor cortex axon terminals increases SNr firing rate ex vivo

**Fig. 4:**
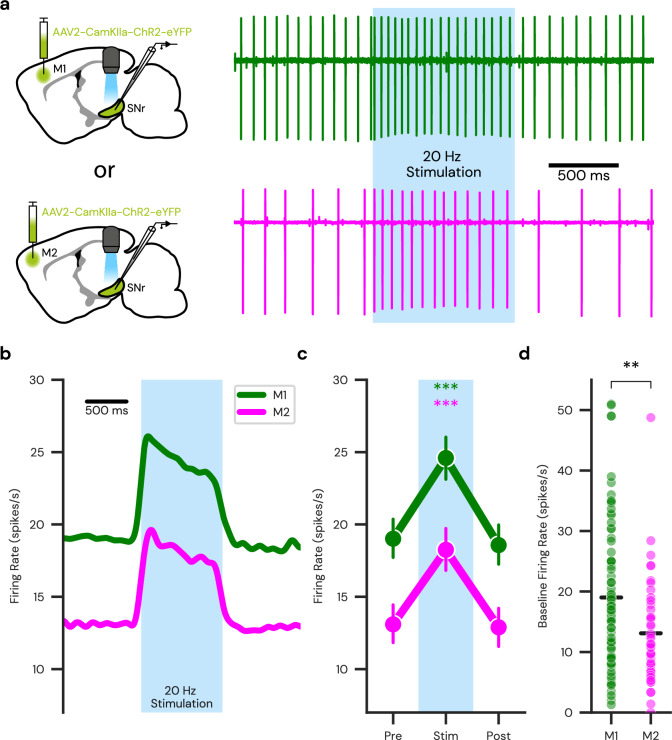
Photoactivation of cortical axon terminals (M1 and M2) increases intrinsic SNr firing. **a** Experimental setup. ChR2 expression was driven in projection neurons of either M1 (upper left) or M2 (lower left) via injection of a viral vector (AAV2-CamkIIa-ChR2-eYFP). SNr neurons were recorded in cell-attached configuration and axon terminals were photostimulated at 20 Hz for one second (right). **b** Mean firing rate response to photostimulation. Instantaneous firing rate was estimated per trial via kernel convolution (Gaussian kernel; σ = 50 ms) before averaging across trials, and across neurons (M1: green, *n* = 79 neurons; *N* = 24 animals; M2: magenta, *n* = 43 neurons, *N* = 16 animals). **c** Binned firing rate during the stimulation period, compared to pre- and post-stimulation periods (M1: green, *n* = 79 neurons; *N* = 24 animals; M2: magenta, *n* = 43 neurons, *N* = 16 animals; ∗∗ *p* < 0.01, ∗∗∗ *p* < 0.001; two-sided Wilcoxon signed-rank test). Data presented as mean ± SEM. **d** Comparison of pre-stimulation firing rate for M1- and M2-recipient neurons (M1: green, *n* = 79 neurons; *N* = 24 animals; M2: magenta, *n* = 43 neurons, *N* = 16 animals; ∗∗ *p* < 0.01; two-sided Mann–Whitney U).

To assess whether cortical input is capable of shaping the firing patterns of SNr neurons, we employed the same viral approach to express ChR2 in either M1 or M2, and photostimulated ChR2-expressing axon terminals while recording the spontaneous activity of SNr neurons in cell-attached configuration (Fig. 4a). In both M1 and M2 conditions, photoactivation at 20 Hz drove a time-locked increase in firing rate, for the duration of the stimulus (M1: *n* = 79 neurons; *N* = 24 animals; M2: *n* = 43 neurons; *N* = 16 animals; Fig. 4a, b). The mean firing rate was significantly increased during the stimulation period relative to both pre- and post-stimulation periods (M1: pre: 19.0 ± 1.3 spikes/s, stimulation: 24.6 ± 1.5 spikes/s, post: 18.6 ± 1.4 spikes/s; pre - stim: *p* < 0.0001; stim - post: *p* < 0.0001; *n* = 79 neurons, *N* = 24 animals; M2: pre: 13.1 ± 1.4 spikes/s, stimulation: 18.2 ± 1.4 spikes/s, post: 12.98 ± 143 spikes/s; pre - stim: *p* < 0.0001; stim - post: *p* < 0.0001; *n* = 43 neurons, *N* = 16 animals; Fig. 4c). Pre- and post-stimulation firing rates were not significantly different in either case (M1: *p* = 0.07; M2: *p* = 0.28). Similarly to what was observed in whole cell configuration, the baseline spontaneous firing rate of M1-responding neurons was higher than that of M2-responding neurons (*p* = 0.005; Fig. 4d). To confirm that the observed excitation of SNr neurons was not due to polysynaptic pathways that may be preserved in a parasagittal brain slice (subthalamonigral projections, for instance), we compared recordings obtained from coronal and parasagittal ex vivo preparations (Supplementary Fig. 5). SNr neuron firing rate was elevated by stimulation of cortical terminals regardless of slice orientation, further confirming a direct activation of the SNr. Thus, optogenetic activation of either M1 or M2 axon terminals can markedly elevate the spiking activity of recipient SNr neurons.

### M1 and M2 corticonigral terminals are topographically organized

**Fig. 5:**
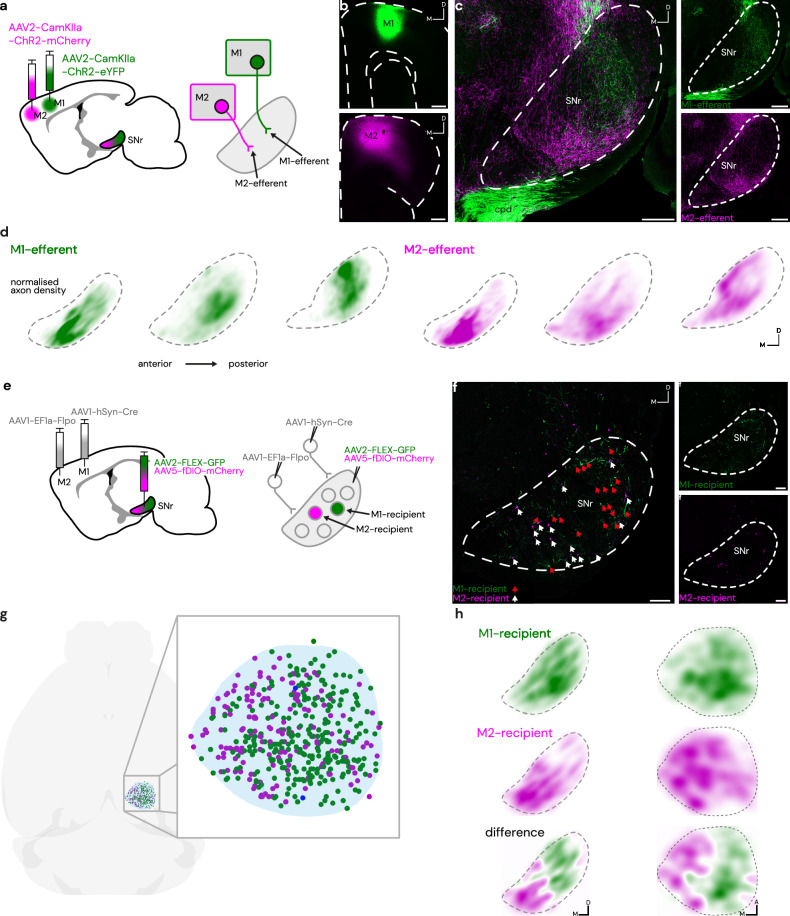
Topographical organization of M1/M2 projections to the SNr. **a** Experimental setup. Simultaneous expression of mCherry and eYFP was driven in projection neurons of M1 and M2 via injection of viral vectors (AAV2-CamkIIa-ChR2-mCherry/AAV2-CamkIIa-ChR2-eYFP). **b** Representative images of injection sites in M1 (upper) and M2 (lower), in a coronal brain slice. **c** Representative confocal image of M1- and M2-efferent axons at the level of the SNr. **d** 2D kernel density estimates of M1 (left; green) and M2 (right; magenta) axons within the SNr in the coronal plane at different positions along the anterior-posterior axis (*N* = 4). **e** Experimental setup. Simultaneous expression of either mCherry or GFP was driven in SNr neurons receiving monosynaptic input from either M1 or M2 via injection of viral vectors (AAV1-hsyn-cre/AAV1-EF1a-flpo/AAV2-FLEX-GFP/AAV5-fDIO-mCherry). **f** Representative confocal image of M1-recipient (green) and M2-recipient (magenta) SNr neurons. **g** Distribution of M1-recipient (green), M2-recipient (magenta), and co-recipient (blue) SNr neurons in 3D.** h** 2D kernel density estimates of M1-recipient (green, upper) and M2-recipient (magenta, lower) neuron locations within the SNr in coronal (left) and horizontal (right) planes (*N* = 3). Scale bars: **b** 500 µm; **c** 200 µm; **f** 200 µm.

Cortical efferents maintain topography as they project caudally through the midbrain and brainstem^27^. To compare the spatial profiles of M1 and M2 axonal innervation within the SNr, we co-expressed distinct fluorophores in M1 and M2 (Fig. 5a, b). At the level of the SNr, M1-efferent axons concentrated in the core of the nucleus, while M2-efferent axons targeted ventromedially, wrapping around the SNr’s ventral borders (Fig. 5c, d, Supplementary Fig. 6). Separation between the two projection patterns was most pronounced in the posterior two-thirds of the nucleus (Fig. 5d). This pattern of innervation is evocative of the laminar parcellation of the SNr proposed by Deniau et al., 1996^54^. This topography, together with the aforementioned differences in electrophysiological properties, suggests that M1 and M2 target distinct populations of SNr neurons.

### M1- and M2-recipient SNr neurons are topographically organized

To further characterize the organization of cortical input to the SNr we employed a transsynaptic viral-labeling approach. We took advantage of the anterograde transsynaptic spread property of the AAV1 serotype to express Cre/Flpo recombinases in neurons postsynaptic to either M1 or M2^55^. We combined this with a second AAV injection targeted to the ipsilateral SNr to selectively express both a Cre-dependent fluorophore (GFP) and a Flpo-dependent fluorophore (mCherry) in neurons receiving monosynaptic cortical input (Fig. 5e, f, Supplementary Fig. 6). We verified the non-dopaminergic identity of putative SNr cells via immuno-labeling for the dopaminergic marker tyrosine hydroxylase (TH; Supplementary Fig. 7). TH-negative, GFP- or mCherry- positive neurons were identified within the SNr, and their soma positions were mapped (Fig. 5g). The spatial distribution of somata was quantified by kernel density estimates in the horizontal and coronal planes (Fig. 5h). Consistent with the observed arrangement of corticofugal terminals, M1- and M2-recipient neurons displayed distinct topographic organization. M1-recipient cells occupy a central core, with M2-recipient neurons lying medially and along the ventral border of the nucleus (Fig. 5h). Only a small proportion of transsynaptically labeled SNr neurons expressed both fluorophores (0.63 ± 0.89% of M1-recipient neurons; 1.39 ± 1.97% of M2-recipient neurons; *N* = 3 animals), indicating that M1- and M2-recipient SNr neurons formed largely non-overlapping populations.

To gain insight into the cortical neurons innervating the SNr, we queried two collections of axonal reconstructions, the Janelia Research Campus MouseLight, and the SEU-Allen Joint Center datasets, for neurons residing in either M1 or M2, and projecting to the SNr (herein referred to as M1_SNr_/M2_SNr_ neurons)^31,56^. As expected for cortical projection neurons, both M1_SNr_ and M2_SNr_ groups demonstrated extensive collateralization throughout the brain, targeting a variety of different subcortical structures, with most terminals located in motor-related subregions of the midbrain, pons and medulla (Supplementary Fig. 8). While not exhaustive, these reconstructions suggest that the cortical signals conveyed to the SNr are broadcast to other regions and may serve to coordinate distinct components of the motor system.

### Functional role of M1- and M2-recipient SNr populations

**Fig. 6:**
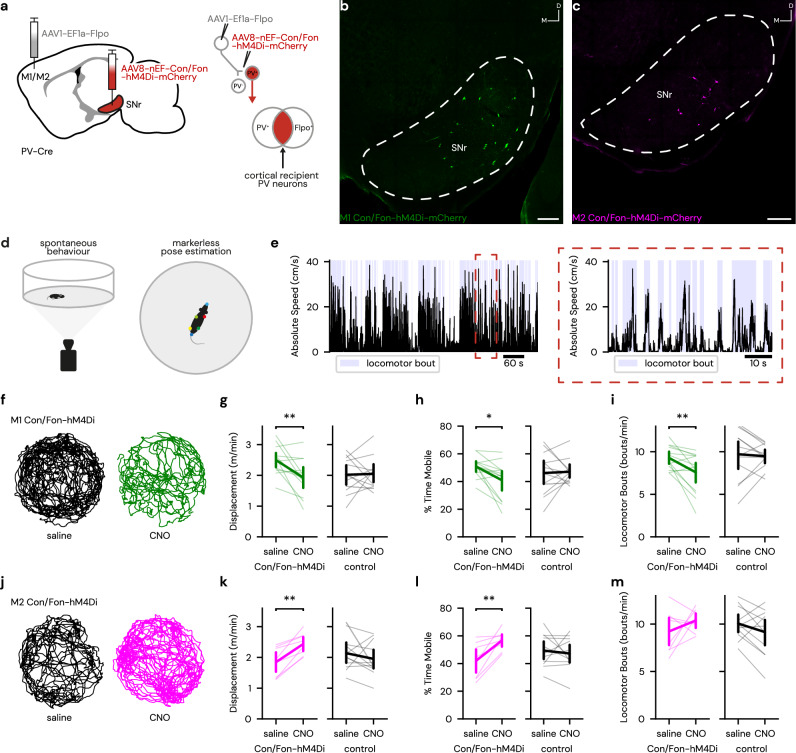
Silencing M1/M2 recipient SNr neurons has contrasting effects on spontaneous locomotion. **a** Experimental setup. AAV1-Ef1a-Flpo was injected bilaterally in either M1 or M2. AAV8-Con/Fon-hM4Di-mCherry was injected bilaterally in the SNr of PV-Cre mice. Anterograde transsynaptic spread drives Flpo expression in postsynaptic neurons, and expression of the inhibitory chemogenetic receptor hM4Di in PV-positive SNr neurons receiving monosynaptic input from the specific cortical region (hM4Di-M1 or hM4Di-M2). For control experiments, AAV1-Ef1a-Flpo was injected in M1/M2, followed by injection of AAV5-nEF-fDIO-mCherry in the ipsilateral SNr of PV-negative littermates. **b** Histological verification of the viral strategy for M1 Con/Fon-hM4Di-mCherry experiments. **c** Histological verification of the viral strategy for M2 Con/Fon-hM4Di-mCherry experiments. **d** Spontaneous behavior was assessed in an open field arena, following intraperitoneal delivery of either Clozapine-N-Oxide (CNO) or saline. Videos were recorded from below, with markerless pose estimation performed offline. **e** Representative locomotor speed trace of a control animal following saline administration. Locomotor bouts identified by blue overlay. Inset: higher magnification of the period identifed by the red dashed box. **f** Representative displacement traces for an M1 Con/Fon-hM4Di animal, following injection of saline (black, **left**) or CNO (green, **right**). **g** Displacement for M1 Con/Fon-hM4Di (green, left, *N* = 13 animals) and control animals (black, right, *N* = 12 animals). **h** Percentage of time spent mobile for M1 Con/Fon-hM4Di (green, left, *N* = 13 animals) and control animals (black, right, *N* = 12 animals). **i** Number of locomotor bouts per minute for M1 Con/Fon-hM4Di (green, left, *N* = 13 animals) and control animals (black, right, *N* = 12 animals). **j–m** Same as (**d**–**g**), for M2 Con/Fon-hM4Di (magenta, *N* = 8 animals) and control animals (black, *N* = 11 animals; ∗ *p* < 0.05, ∗∗ *p* < 0.01, ∗∗∗ *p* < 0.001; two-tailed paired *t* test). Data presented as mean ± SEM. Scale bar: **b**,** c**: 200 µm.

To probe the functional role of cortically-targeted SNr neurons without interfering with collateral targets, we employed an intersectional transsynaptic viral strategy. As the majority of GABAergic SNr neurons express parvalbumin (PV)^4^, we used PV-Cre mice to gain selective access to the GABAergic population of the SNr. We injected AAV1-Flpo bilaterally in either M1 or M2, to transsynaptically express Flpo in cells postsynaptic to the target region. This was combined with an injection of a Cre-and-Flpo-dependent inhibitory chemogenetic receptor (hM4Di) targeted to the SNr. This approach drove expression of the inhibitory chemogenetic receptor hM4Di exclusively in PV-positive (PV + ) SNr neurons receiving monosynaptic cortical input (Fig. 6a–c, Supplementary Fig. 9). Spontaneous behavior was assessed in an open field context (Fig. 6d, e). Silencing M1-recipient PV+ SNr neurons via intraperitoneal injection of the hM4Di agonist Clozapine-N-oxide (CNO) induced a decrease in total displacement the open field (Con/Fon-hM4Di: −0.57 ± 0.6 m/minute; N = 13 animals; control: +0.03 ± 0.63 m/minute; N = 12 animals; Fig. 6f-g). This was not due to impaired locomotor capacity but rather attributable to an increase in immobility and a decrease in locomotor bout initiations (Con/Fon-hM4Di: −10.0 ± 11.3 % time mobile; −1.69 ± 1.96 locomotor bouts/minute; N = 13 animals; control: +0.94 ± 14.3 % time mobile; −0.23 ± 2.14 locomotor bouts/minute; N = 12 animals; Fig. 6h-i, Supplementary Fig. 10, Supplementary Movies 1, 2). Silencing M1-recipient PV+ SNr neurons increased the overall frequency of stationary, non-locomotor behaviors. These episodes included stationary behaviors both with, and without, head movements (Supplementary Fig. 10, Supplementary Movies 3, 4). In contrast, chemogenetic silencing of the M2-recipient SNr population prompted an increase in total displacement and time spent mobile (Con/Fon-hM4Di: + 0.59 ± 0.31 m/minute; + 14.7 ± 10.3 % time mobile; N = 8 animals; control: −0.18 ± 0.61 m/minute; −2.1 ± 12.6 % time mobile; N = 11 animals; Fig. 6j-m, Supplementary Movies 5, 6). Silencing M2-recipient SNr neurons led to a decrease in the observed frequency of stationary episodes, with a notable increase in locomotor behaviors (Supplementary Fig. 10). These results suggest contrasting behavioral roles for the SNr populations targeted by M1 and M2.

## Discussion

To understand cortical control of downstream motor centers requires a thorough account of descending connectivity. Similarly, to fully understand how SNr activity is regulated, a complete description of SNr inputs is necessary. In this study, we have characterized direct, functional connections from M1 and M2 to SNr, by combining virally targeted optogenetics, ex vivo electrophysiology, and behavioral perturbations of postsynaptic neurons. Our results show that subpopulations of GABAergic SNr neurons receive monosynaptic, glutamatergic input from the primary and secondary motor cortices and demonstrate that this input is capable of shaping SNr neuron firing patterns.

### Distinct properties of M1-SNr and M2-SNr pathways

We have shown that neurons of both M1 and M2 innervate GABAergic SNr neurons, however, the two pathways differ in several aspects. SNr neurons receiving inputs from M1 displayed different electrophysiological properties from M2-recipient neurons, with higher spontaneous spiking frequency and lower spike thresholds (Figs. 2g, 4d). M1 and M2 projections target different (although partially overlapping) regions within SNr, with very little convergence onto individual SNr neurons (Fig. 5). Chemogenetic inhibition of M1- and M2-recipient SNr neurons produced opposing effects on spontaneous behavior (Fig. 6). These results, together with the selectivity observed at the presynaptic (lack of inputs from the primary somatosensory cortex) and postsynaptic (sparse inputs to dopaminergic neurons of the substantia nigra) levels, suggest that corticonigral projections are organized into distinct functional modules. This apparent specialization agrees with previous reports of target-defined SNr subpopulations differing in their topography, electrophysiology, and molecular properties^4,12^. In this study we focused on corticonigral projections from only two cortical regions (M1 and M2), while demonstrating the lack of such innervations from S1 (Supplementary Fig. 3). It is, however, possible that other cortical regions also project to the SNr, as has recently been shown for insular cortex^57^.

### Cortical input to the basal ganglia

Information flow through the basal ganglia has traditionally been thought of in terms of competing pathways^58–62^. Striatal neurons of both the direct and indirect pathways receive robust input from both cortical hemispheres^41,63–66^. They, in turn, transmit signals to the SNr either monosynaptically or polysynaptically, via the GPe. Moreover, most direct pathway striatal neurons also send collaterals to the GPe^67–69^. Cortical commands are, therefore, differentially propagated to the SNr via these multisynaptic routes.

The disynaptic hyperdirect (cortex-STN-SNr) pathway provides excitatory input to SNr neurons via glutamatergic neurons in the STN^62,70^. This excitatory input drives increased SNr firing and ultimately elicits increased inhibition of both thalamic nuclei and downstream motor targets. The hyperdirect pathway therefore provides a route for a cortical command to either suppress competing motor programs through inhibition of downstream motor centers or interrupt ongoing movement via the SNr-thalamus-cortex loop^70,71^. Both axonal reconstructions and intersectional retrograde labeling studies in mice have demonstrated that some M1/M2 neurons innervating the SNr also innervate the STN (Supplementary Fig. 8)^15,27,29^. Whether individual SNr neurons receive concurrent excitation from both STN and motor cortex has not been explicitly shown, although all available evidence suggests that all SNr neurons receive STN input^44^. Cortical projections also show a topographic arrangement within the STN, and some STN-targeting cortical axons collateralize in the SNr^29^. Although STN input is a powerful driver of SNr neurons, STN neurons are also subject to competitive inhibition from the prototypical neurons of the GPe. A functional advantage of a monosynaptic excitatory pathway may be that a cortical command could reach the SNr unperturbed, providing robust excitatory drive independently of concurrent activity in the other basal ganglia structures. However, many SNr-projecting cortical neurons do not collateralize within the STN, and vice versa–suggesting that distinct cortical commands can be independently conveyed to each nucleus.

Cortical projections also target the globus pallidus externa and interna, both of which complement the SNr as elements of the basal ganglia output stage^30,57,72–74^. GPe-projecting cortical neurons show diverse projection patterns, with some neurons collateralizing in the SNr. One electrophysiological study in rats found that, within the GPe, input from motor cortices was biased towards the striatum-projecting arkypallidal neurons, which do not project to the SNr^30^. In contrast, multiple studies in mice have reported cortical input to the SNr-projecting prototypic neurons of the GPe, as well as the arkypallidal cells^29,57,74^. Corticopallidal projections may serve a similar function as corticonigral projections: bypassing the striatum for direct access to basal ganglia outputs.

While all SNr-projecting cortical neurons appear to originate in layer 5, both M1 and M2 contain a diversity of unique projection neuron-populations that are activated dynamically in behavioral contexts^32,75–77^. Non-specific experimental activation of M1 or M2 in the intact brain would conceivably excite all basal ganglia structures. Electrical stimulation of the rodent motor cortex in vivo generates multiphasic responses in SNr neurons, illustrating the multiple descending routes of information flow through the basal ganglia^78,79^. As we have shown that corticonigral input is monosynaptic, the time delay between cortical activation and SNr response would be minimal. Thus, the early excitation phase commonly attributed to the hyperdirect pathway may also reflect monosynaptic input from the corticonigral pathway. This could explain the persistence of an early excitatory response in SNr neurons of mice following selective ablation of the hyperdirect pathway^70^.

### Cortical coordination of the motor system

Monosynaptic cortical input to the SNr could be reflective of a broader circuit motif for cortical orchestration of the brain’s motor system. By simultaneously activating multiple downstream structures, projection neurons of the motor cortex broadcast motor commands to all relevant targets together^80,81^. Certain neurons of the motor cortex exhibit increased activity preceding motor execution^82,83^. This type of command could excite SNr neurons in anticipation of forthcoming signals from, for example, the striatum. By reinforcing an SNr neuron’s firing rate, cortical commands may prevent premature release of motor programs and/or suppression of competing ones, enhancing coordination between components of the motor system. Input arriving directly from the cortex would provide higher temporal fidelity than signals that are first processed in upstream basal ganglia nuclei. The ability to perform complex movements with high temporal precision, or to respond quickly to a changing environment, may depend on this unhindered communication between the cortex and subcortical targets.

We report that a large fraction of recorded SNr cells responded to photostimulation of M1/M2 axon terminals. This number may represent an overestimation, as recordings were obtained selectively from SNr neurons in the vicinity of fluorescent corticonigral axons. However, even within these axonal fields there were SNr neurons that did not receive synaptic input, supporting selective targeting of specific SNr subpopulations. Signals with high temporal precision—i.e., monosynaptic cortical input—would logically be targeted towards the SNr neurons participating in motor circuits supporting high temporal fidelity actions. M1 neurons that innervate SNr were shown to be located in motor regions relating primarily to the control of the forelimbs and mouth^15,27^. This is complemented by the higher density of M1 axon innervation observed in the dorsolateral regions of the SNr. The medial SNr has been implicated in associative functions and behavioral state transitions, while neurons along the ventral border of the nucleus are critical for locomotor control^19,84^. Medially located neurons fire at lower rates than their lateral counterparts, and project to different targets in the brainstem^4^. Relative to their lateral counterparts, medial SNr neurons receive increased input from hypothalamic and state-related midbrain regions, and are involved in the processing of slower homeostatic signals^19^.

### Differential behavioral roles of M1/M2 recipient SNr-PV neurons

In the behavioral experiments presented here (Fig. 6 and Supplementary Fig. 9-10), we chose to manipulate PV-expressing neurons receiving input from either M1 or M2. The vast majority of GABAergic neurons in the SNr express PV, while the rest of the GABAergic SNr neurons share similar projection patterns^4^. Performing in vivo manipulations in an intersectional manner with PV-Cre mice was motivated so as to avoid the potential manipulation of other neuron types in the vicinity of the SNr, most notably the dopaminergic populations of either the SNr or SNc (Supplementary Fig. 1, 7).

We chemogenetically inhibited M1- and M2- recipient neurons and observed constrasting behavioral changes in response to the respective manipulations. While this approach does not allow for specific manipulation of the corticonigral terminals onto SNr cells, it did show that manipulation of PV-positive M1- and M2-recipient SNr neurons differentially affected behavior. Our anatomical data demonstrates that the M1/M2 recipient populations in SNr are topographically organized. This is consistent with the topographical organization of other SNr afferents - as well as the topography in SNr output organization—shown in previous studies^4,11,15,27,44^. The highly organized structure of these circuits through the SNr suggests a system of parallel information processing channels for discrete functional roles. The contrasting behavioral impact we observed when manipulating distinct input-defined SNr subpopulations further supports this.

The decrease in locomotion observed when silencing M1-recipient neurons might appear contrary to the generally held view of the basal ganglia’s permissive role in motor control (wherein decreased SNr activity should allow motor execution).

Hindbrain-projecting cortical neurons have been shown to be specifically implicated in the control of discrete body parts^24,27,81^ Therefore, we reason that M1-recipient SNr neurons could be involved in coordinating discrete movements, rather than whole-body movements. By silencing these inhibitory neurons such movements may be promoted – to the exclusion of locomotion. In rodents, the capacity for spontaneous locomotion is largely spared by cortical lesions and is likewise insensitive to optogenetic M1 inactivation^85,86^. Primary motor cortex is, however, required for flexible visuomotor coordination, in which corticonigral projections could play a role.

In contrast, inhibition of the M2-recipient SNr population induced increased locomotion - as expected from the canonical basal ganglia model - suggesting that this SNr population is directly involved in locomotor control. Indeed, the subpopulation of SNr neurons primarily implicated in locomotor control lies to the ventromedial extreme of the nucleus, partially overlapping with the M2-recipient populations that we labeled^84^.

In conclusion, our results demonstrate the existence of a direct access point from which different cortical motor areas can affect activity in the substantia nigra pars reticulata - the basal ganglia’s output stage.

## Methods

### Materials availability

This study did not generate new unique reagents.

### Experimental model and subject details

All experiments were performed with approval of the local ethical board, Stockholm Norra Djurförsöksetiska Nämnd, under an ethical permit to Gilad Silberberg (N2022-2020). Both male and female wild type mice (C57BL/6 J, stock #000664, The Jackson Laboratory, U.S.A.), and heterozygous PV-Cre mice (stock #000664, The Jackson Laboratory, U.S.A.; maintained on a C57BL/6 J background) were used in this study. Mice were housed in groups of up to five and maintained on a 12-h light cycle with ad libitum access to standard food and water. Animal rooms were kept between 20 and 22 °C, and 50–65% humidity.

### Method details

#### Surgeries

Mice, aged between six and ten weeks, were anesthetized with isoflurane (VM Pharma AB, Sweden), and placed in a stereotaxic frame (Stoelting, U.S.A). Craniotomies were drilled with the following coordinates relative to bregma: M1 (AP: 1.5 mm, ML: −1.75 mm, DV: −0.8 mm), M2 (AP: 2.3 mm, ML: −1.00 mm, DV: −0.9 mm), SNr (AP: −3.4 mm, ML: −1.5 mm, DV: −4.5 mm). For electrophysiological experiments 0.4 µL of AAV2-CaMKIIa-hChR2(H134R)-eYFP (Addgene #26969; Penn Vector Core, U.S.A.) was injected into either M1 or M2. For dual-channel anterograde tracing experiments AAV2-CaMKIIa-hChR2(H134R)-eYFP (as above) and AAV5-CaMKIIa-hChR2(H134R)-mCherry (Addgene #26975; Penn Vector Core, U.S.A.) were injected into M1 and M2 (coordinates and volumes as above) in the same surgery. For transsynaptic labeling experiments AAV1-hSyn-Cre (Addgene #105553; Penn Vector Core, U.S.A.) and AAV1-Ef1a-Flpo were injected into M1 and M2. A mixture of AAV2-pCAG-FLEX-eGFP (Addgene #51502; Penn Vector Core, USA), and AAV5-Ef1a-fDIO-mCherry (Addgene #114471; Penn Vector Core, U.S.A.) was injected into the SNr during the same surgery (coordinates and volumes as above). For chemogenetic experiments, AAV1-Ef1a-Flpo (Addgene #55637; Penn Vector Core, U.S.A.) was injected bilaterally into either M1 or M2 of heterozygous PV-Cre mice. AAV8-nEF-Con/Fon-DREADD-Gi-mCherry (Addgene #177672; Penn Vector Core, U.S.A.) was injected bilaterally in the SNr. For control experiments, the SNr-targeted injections were substituted with AAV5-Ef1a-fDIO-mCherry (as above), in PV-negative littermates. For all injections, viruses were injected using a micropipette, at a rate of 0.1 µL/min (Quintessential Stereotaxic Injector, Stoelting, U.S.A.). The pipette was held in place for at least 5 min before being slowly retracted from the brain. Buprenorphine (Indivior Europe Limited; Apoteket, Sweden) was administered perioperatively (0.1 mg/kg).

#### Brain slice preparation

Mice were sacrificed at least three weeks following virus injections. Mice were anesthetized with isoflurane (VM Pharma AB, Sweden) and decapitated. Brains were removed while immersed in ice-cold cutting solution containing the following (in mM): 2.5 KCl, 1.25 NaH_2_PO_4_, 0.5 CaCl_2_, 7.5 MgCl_2_, 10 glucose, 25 NaHCO_3_, 205 sucrose. Either coronal or parasagittal slices were cut at a thickness of 250 µm with a Leica VT1200S Vibratome (Leica Microsystems GmbH, Germany). Slices were incubated for 30 min at 35 °C in a chamber filled with artificial cerebrospinal fluid (aCSF) saturated with 95% oxygen and 5% carbon dioxide. ACSF contained the following (in mM): 125 NaCl, 25 glucose, 25 NaHCO_3_, 2.5 KCl, 2 CaCl_2_, 1.25 NaH_2_PO_4_, 1 MgCl_2_. Before recording, slices were kept in aCSF at room temperature for at least 45 min.

#### Ex vivo recordings

Whole-cell patch clamp recordings were obtained in oxygenated aCSF, which was continuously perfused throughout the experiment and maintained at 35 °C with a temperature control unit (Luigs and Neumann GmbH, Germany). Neurons were visualized using infrared differential interference contrast (IR-DIC) microscopy on a BX51WI (Olympus, Japan) upright microscope, with a ×40 long-working-distance immersion objective and a digital camera (Hamamatsu Photonics, Japan). Borosilicate glass pipettes were pulled using a P1000 micropipette puller (Sutter Instrument, U.S.A.) to a resistance of 6–8 MΩ and filled with an intracellular solution containing (in mM): 130 K-gluconate, 5 KCl, 10 HEPES, 4 Mg-ATP, 0.3 GTP, 10 Na_2_-phosphocreatine. For post-hoc localization and morphological analysis, 0.2% neurobiotin (Vector Laboratories, U.S.A.) was added to the intracellular solution in a subset of experiments. Liquid junction potentials (measured at approximately 11 mV) were not corrected for. For experiments conducted in voltage clamp configuration, a cesium-based internal solution was used instead, and contained (in mM): 10 CsCl, 110 CsMeSO_3_, 10 HEPES, 10 Na_2_-Phosphocreatine, 4 ATP-Mg, 0.3 GTP-Na, 10 TEA-Cl, 1 QX-314-Cl. To assess the presence of calcium-permeable AMPA receptors, a polyamine-containing intracellular solution was used, containing (in mM): 105 CsMeSO_3_, 8 NaCl, 10 HEPES, 4 MgATP, 0.3 NaGTP, 10 Na_2_-phosphocreatine, 0.3 EGTA, 5 TEA-Cl, 5 Qx-314, and 0.1 spermine. The liquid junction potential for this intracellular solution was measured at approximately 20 mV and was corrected for. Up to three cells were recorded simultaneously. Recordings were performed with MultiClamp 700B amplifiers (Molecular Devices, U.S.A.), filtered at either 4 kHz (voltage clamp) or 10 kHz (current clamp), and digitized (10–20 kHz) via an ITC-18 (HEKA Elektronik, U.S.A.) acquisition board. Recordings were acquired with IGOR Pro 6.37 (WaveMetrics, U.S.A.).

SNr neuron identity was confirmed by characteristic electrophysiology. Intrinsic neuronal properties were extracted in whole cell current clamp configuration using a standardized series of hyperpolarizing and depolarizing current injections. A baseline hyperpolarizing current was injected to maintain resting membrane potentials at approximately −75 mV. To activate ChR2, 473 nm light was transmitted to the slice via the 40x objective (1 mW; CoolLED Limited, U.K.). Trains of light pulses (2 ms duration, 8 pulses) were delivered at 10, 20 and 40 Hz.

For voltage clamp experiments cells were held at –70 mV. A single light pulse was delivered to characterize EPSCs. In all conditions responses were averaged from a minimum of 5 sweeps, with a 10 s interval between consecutive sweeps. In a subset of experiments, light stimulation was delivered at 20 Hz for 1 s while holding the cell in cell-attached configuration, before moving to whole-cell configuration. In all experiments, the light-delivering objective was centered above the recorded neurons in the SNr. Pharmacological agents were bath applied to the slice, via the perfusate. These included tetrodotoxin (TTX; 1 μM; Tocris, U.K., Cat: 1069) and 4-aminopyridine (4-AP; 100 μM; Tocris, U.K., Cat: 0940) to isolate monosynaptic responses, NBQX disodium salt (NBQX; 10 μM; Tocris, U.K., Cat: 1044) and D-APV (50 μM, Tocris, U.K., Cat: 0106), to selectively block glutamatergic signaling, and gabazine hydrobromide (SR-95531; gabazine 10 μM; Sigma-Aldrich, U.K., Cat: 1262) to selectively block GABA_A_ receptor signaling.

For morphological analysis, 0.2% neurobiotin (Vector Laboratories, U.S.A., Cat: SP-1120) was added to the intracellular solution in a subset of experiments. The recorded slices were fixed overnight in 4% (w/v) paraformaldehyde (PFA) with picric acid, washed in 0.01 M phosphate buffered saline (PBS, pH 7.3), and incubated for 48–72 h at 4 °C with Cy5-conjugated streptavidin (1:1000, Jackson ImmunoResearch Laboratories) in 0.01 M PBS containing 0.6% Triton X-100. Following three washes in PBS, slices were counterstained with DAPI (1:5000; Sigma-Aldrich, Cat: D9542) and mounted on Superfrost Plus microscope slides (Thermo Fisher Scientific, U.S.A.) with ProLong Antifade mounting agent (Thermo Fisher Scientific, U.S.A.). Confocal z-stacks were taken on either a Zeiss LSM 700 or LSM 800 microscope, running ZEN Blue (version 2.1; Zeiss, Germany). Neuron morphologies were reconstructed in a semi-automated manner, via neuTube^87^.

#### Histology

For viral tracing experiments, animals were anesthetized and transcardially perfused with 4% PFA in 0.01 M PBS, at least three weeks after virus injections. Brains were removed and post-fixed overnight in PFA at 4 °C. Brains were then transferred to a sucrose/PBS solution (30% w/v) for 18–24 h. Serial 50 µm coronal or sagittal sections were cut on a cryostat. To distinguish GABAergic from dopaminergic neurons, certain sections were stained for tyrosine hydroxylase (TH) immunoreactivity. Sections were incubated in blocking solution (5% normal donkey serum and 0.3% Triton X-100 in PBS; Sigma-Aldrich, U.S.A.) for 30 min and then incubated in rabbit anti-TH polyclonal antibody (Sigma-Aldrich, U.S.A.; Cat: AB152) diluted 1:1000 in 0.3% Triton X-100 in PBS, at 4 °C, overnight. For dual–channel anterograde experiments, mCherry signal was amplified with a rabbit anti-mCherry polyclonal antibody (Abcam, U.K.; Cat: ab167453). Sections were rinsed in PBS and incubated in either Cy3-conjugated goat, or Cy5-conjugated donkey, anti-rabbit polyclonal secondary antibody (The Jackson Laboratory, U.S.A.; Cat: 111-165-003 and 705-165-147) for two hours at room temperature. Sections were stained with DAPI (1:5000; Sigma-Aldrich, U.S.A.; Cat: D9542) and mounted with ProLong Antifade mounting agent (Thermo Fisher Scientific, U.S.A.). Confocal tile scans were taken on a LSM 800 microscope, running ZEN Blue (version 2.1; Zeiss, Germany). Complimentary widefield images were taken with an Olympus XM10 digital camera mounted on an Olympus BX51 fluorescence microscope (both Olympus Sverige AB, Sweden). Image brightness and contrast were adjusted in FIJI (version 2.16.0)^88^. Sections were registered to the Allen Mouse Brain Common Coordinate Framework (version 3) using DMC-BrainMap (version 0.1.7)^89^. Axon density was assessed by binarizing images. For mapping of soma position, only TH-negative neurons within the boundaries of the SNr were counted.

#### Behavioral experiments

Open field assays were conducted in a 35 cm diameter circular arena with a transparent acrylic floor. Animals were habituated to the arena through 3 sessions of 15–20 min, over 3 days, prior to testing. Clozapine-N-Oxide (CNO; Tocris, U.K., Cat: 6329) was dissolved in saline. Animals were randomly assigned either CNO (1 mg/kg bodyweight) or a saline vehicle (equivalent volume) on the first day, with the converse being given on the second day. These were administered via intraperitoneal injection. 30 min after administration, animals were moved to the open field arena. Spontaneous behavior in the open field was recorded for 10 min per session. Videos were recorded from below, with a machine vision camera operating at 50 fps (Teledyne FLIR, U.S.A.). Acquisition was performed via Spinnaker SDK (version 4.2.0.83; Teledyne FLIR, U.S.A.). Tests were separated by 24 h. After behavioral tests, animals were sacrificed for histological confirmation, following the same method detailed above. In a subset of animals, chemogenetic activity was confirmed via post-hoc ex vivo electrophysiology. Acute slices were prepared as detailed above. After initial characterization of electrophysiological properties, CNO (10 µm) was bath-applied via the perfusate. Following ex vivo electrophysiology, slices were also assessed histologically for target specificity. Individuals were excluded from further analysis if mCherry-positive neurons were found external to the SNr, or if no mCherry-positive neurons were found within the SNr.

#### Behavioral Analysis

Pose estimation was performed via DeepLabCut (version 3.0.0)^90^. Keypoints with confidence below 0.6 were omitted and linearly interpolated. Nose, forepaws, hindpaws and tailbase were tracked - the tailbase was used for primary analysis. A locomotor bout was defined by a velocity threshold of 2 cm/s and a duration threshold of 250 ms.

For further analysis, DeepLabCut pose estimations were used to train a state-space model (Keypoint-Moseq; version 0.6.7)^91^. Briefly, DeepLabCut keypoints were converted to egocentric coordinates, and transformed via principal component analysis (four components). An autoregressive hidden Markov model was fitted to the resulting data. All hyperparameters were left to Keypoint-Moseq defaults. 71 behavioral syllables were identified and organized via hierarchical clustering (Supplementary Fig. 10). Clusters were manually labeled with coarse semantic descriptions based on video slicing by individual syllables, in conjunction with analysis of egocentric coordinate distribution. Syllable usage frequency was calculated and normalized for each session.

#### Axonal reconstruction analysis

Two datasets of morphological reconstructions (Janelia Mouselight: http://ml-neuronbrowser.janelia.org/ and SEU-Allen: https://braintell.org/projects/fullmorpho/) were queried to identify single axons originating in either M1 or M2, with axonal terminals located within the SNr. 27 individual morphologies (Mouselight: 13, SEU-Allen: 14) meeting these criteria were further examined to identify other target structures. To this end, OBJ files representing the 3D geometry of all Allen Mouse Brain Common Coordinate Framework structures were obtained via the Allen Software Development Kit (https://allensdk.readthedocs.io/). Structures were grouped into mesoscale regions based on the Allen Institute’s ontology. Several further morphologies were found to have axons passing through the SNr without branching or terminating. Although such axons could have en passant synaptic boutons within the SNr, they were not considered for the present analysis. Where necessary, mediolateral coordinates were mirrored, such that only the right hemisphere was visualized.

#### Quantification and statistical analysis

Neuron distribution was quantified using DMC-BrainMap (version 0.1.7)^89^. Distributions and morphological reconstructions were visualized with Blender 4.0. All other analysis was performed using custom written Python scripts (Python 3.9/3.11), using open-source packages from eFEL (version 5.6.25), Elephant (version 1.2), and NEO (version 0.14.0)^92–94^. Results are presented in the text as mean ± SEM. Normality was assessed for all distributions via Shapiro-Wilk tests. Statistical significance is defined as ∗ *p* < 0.05, ∗∗ *p* < 0.01, and ∗∗∗ *p* < 0.001. Statistical analysis was performed with the open-source package scipy (version 1.15.2). Neuron numbers are denoted in text by ‘n’, while animal numbers are denoted by ‘N’.

### Reporting summary

Further information on research design is available in the Nature Portfolio Reporting Summary linked to this article.

## Supplementary information

Supplementary Information

Description of Additional Supplementary files

Supplementary Movie 1

Supplementary Movie 2

Supplementary Movie 3

Supplementary Movie 4

Supplementary Movie 5

Supplementary Movie 6

Reporting Summary

Transparent Peer Review file

## Source data

Source Data

## Data Availability

A source data file supporting the findings of this study is provided with this paper. Further data will be made available upon request. Source data are provided with this paper.

## Code availability

Visualization code is available at https://github.com/wstho/brainblenda. Additional code used in this study will be made available upon request.
