# Supplementary material for: Motor cortex directly excites the substantia nigra pars reticulata, the basal ganglia output nucleus
Source: Nat Commun. 2026 Jun 23;17:5551. doi: 10.1038/s41467-026-74569-w (PMC13291323; doi:10.1038/s41467-026-74569-w)
Supplement: Supplementary file 1 — Supplementary Information [file 41467_2026_74569_MOESM1_ESM.pdf]

Motor cortex directly excites the substantia nigra *pars reticulata*, the basal ganglia output nucleus

William Scott Thompson[1], Patryk Wekwejt[1], Sten Grillner*[1] and Gilad Silberberg*[1]

Supplementary information containing 10 figures and legends.

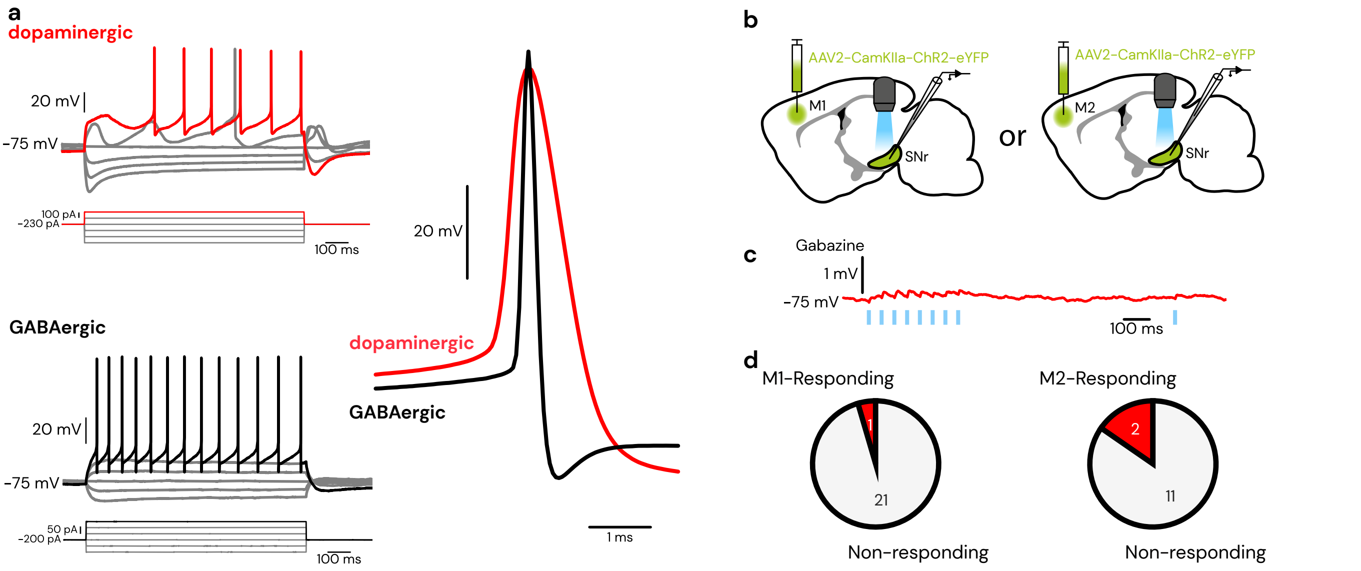
a
dopaminergic
20 mV
-75 mV
100 pA
-230 pA
100 ms
20 mV
dopaminergic
GABAergic
1 ms
GABAergic
20 mV
-75 mV
50 pA
-200 pA
100 ms
b
AAV2-CamKIIa-ChR2-eYFP
M1
SNr
or
AAV2-CamKIIa-ChR2-eYFP
M2
SNr
c
Gabazine
1 mV
-75 mV
100 ms
d
M1-Responding
1
21
Non-responding
M2-Responding
2
11
Non-responding

Supplementary Fig. 1: Dopaminergic neurons within the SNr are electrophysiologically distinguishable from GABAergic neurons

a: Electrophysiological characterization of dopaminergic and GABAergic neurons in the substantia nigra. Left: representative responses to current steps for a dopaminergic (upper) and GABAergic (lower) neuron. Right: representative single action potentials for a dopaminergic (red) and GABAergic (black) neuron. **b**: Experimental setup. ChR2 expression was driven in projection neurons of either M1 or M2 via injection of a viral vector (AAV2-CamkIIa-ChR2-eYFP). SNr neurons were recorded in acute brain slices via whole-cell patch clamp, in current clamp configuration. **c**: EPSPs were recorded in response to trains of photostimulation (2 ms, 1 mW; stimulation onset indicated by blue marks) during bath application of the $GABA_A$ antagonist gabazine (10 μM). Hyperpolarizing current was injected to keep the baseline membrane potential at approximately -75 mV. **d**: Proportion of recorded dopaminergic neurons that responded to photostimulation; numbering represents total counts (M1: n = 1 of 22 neurons; N = 14 animals; M2: n = 2 of 13 neurons; N = 6 animals).

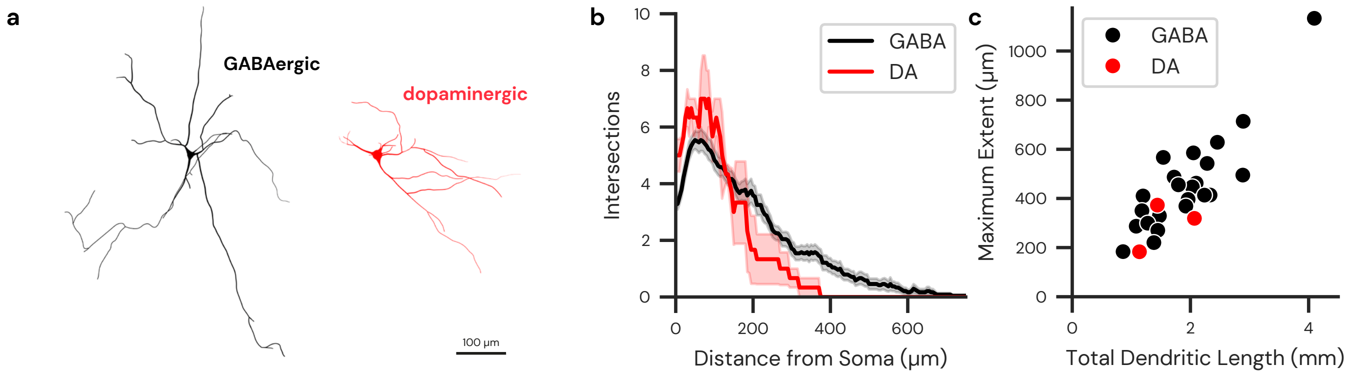

a
GABAergic
dopaminergic
100 µm
b
Intersections
Distance from Soma (µm)
GABA
DA
0
2
4
6
8
10
0
200
400
600
c
Maximum Extent (µm)
Total Dendritic Length (mm)
GABA
DA
0
200
400
600
800
1000
0
2
4

Supplementary Fig. 2: Morphological properties of recorded neurons

a: Representative reconstructions of recorded GABAergic SNr (black, left) and dopaminergic (red, right) neurons. **b**: Sholl analysis of reconstructed neurons (GABAergic: n = 24 neurons; dopaminergic: n = 3 neurons). SEM indicated by error band. **c**: Scatterplot of total dendritic length and maximum dendritic extent from the soma for GABAergic (black) and dopaminergic (red) reconstructions (GABAergic: n = 24 neurons; dopaminergic: n = 3 neurons).

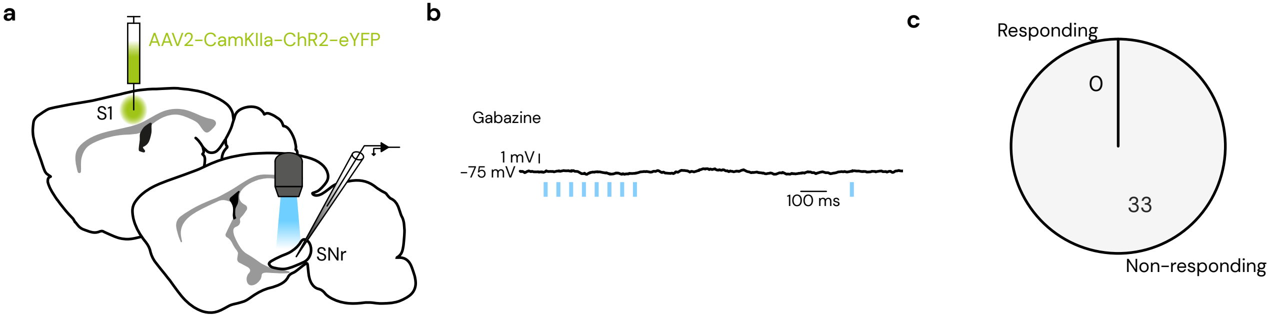
a
AAV2-CamKIIa-ChR2-eYFP
S1
SNr
b
Gabazine
1 mV
-75 mV
100 ms
c
Responding
0
33
Non-responding

Supplementary Fig. 3: Photostimulation of S1 axon terminals does not yield excitation of SNr neurons

a: Experimental setup. ChR2 expression was driven in projection neurons of S1 via injection of a viral vector (AAV2-CamkIIa-ChR2-eYFP). SNr neurons were recorded in acute brain slices via whole-cell patch clamp, in current clamp configuration. **b**: No EPSPs were observed in response to trains of photostimulation (2 ms, 1 mW; stimulation onset indicated by blue marks) during bath application of the $GABA_A$ antagonist gabazine (10 μM). Hyperpolarizing current was injected to keep the baseline membrane potential at approximately -75 mV. **c**: Proportion of recorded neurons that responded to photostimulation; numbering represents total counts (n = 0 of 27 neurons; N = 4 animals).

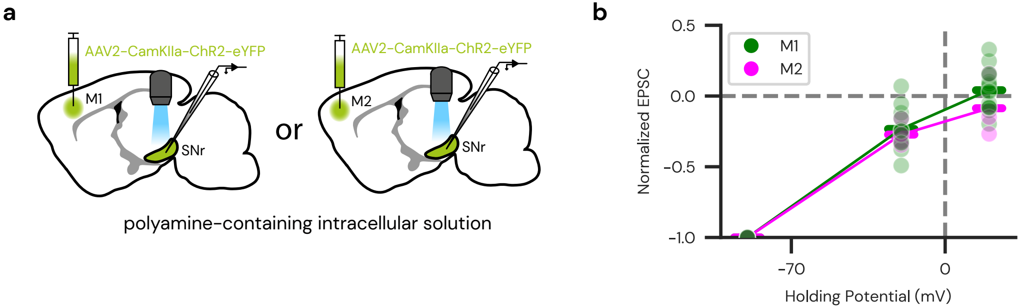

a
AAV2-CamKIIa-ChR2-eYFP
M1
SNr
or
AAV2-CamKIIa-ChR2-eYFP
M2
SNr
polyamine-containing intracellular solution
b
M1
M2
0.5
0.0
-0.5
-1.0
Normalized EPSC
-70
0
Holding Potential (mV)

Supplementary Fig. 4: Polyamine-dependent rectification of AMPA receptors at corticonigral synapses

a: Experimental setup. ChR2 expression was driven in projection neurons of either M1 or M2 via injection of a viral vector (AAV2-CamkIIa-ChR2-eYFP). SNr neurons were recorded in acute brain slices via whole-cell patch clamp, in voltage clamp configuration with a polyamine-containing intracellular solution. **b**: Summary of optogenetically evoked EPSCs across holding potentials (M1: n = 15 neurons, N = 4 animals; M2: n = 4 neurons, N = 3 animals).

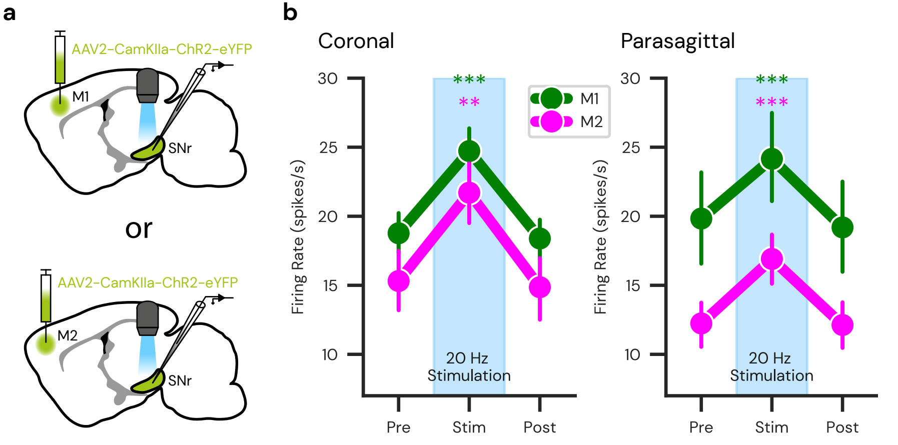
a
AAV2-CamKIIa-ChR2-eYFP
M1
SNr
or
AAV2-CamKIIa-ChR2-eYFP
M2
SNr
b
Coronal
Parasagittal
***
**
***
***
M1
M2
Firing Rate (spikes/s)
30
25
20
15
10
20 Hz
Stimulation
Pre
Stim
Post

Supplementary Fig. 5: Effect of slice orientation on cell-attached firing rates

a: Experimental setup. ChR2 expression was driven in projection neurons of either M1 (upper left) or M2 (lower left) via injection of a viral vector (AAV2-CamkIIa-ChR2-eYFP). SNr neurons were recorded in cell-attached configuration and axon terminals were photostimulated at 20 Hz for one second. **b:** Binned firing rate during the stimulation period, compared to pre- and post-stimulation for neurons recorded from coronal (left; M1: n = 61 neurons; M2: n = 18 neurons) and parasagittal (right; M1: n= 12 neurons; M2: n= 31 neurons) slices (** $p < 0.01$, *** $p < 0.001$; two-tailed paired t-test). Data presented as mean ± SEM.

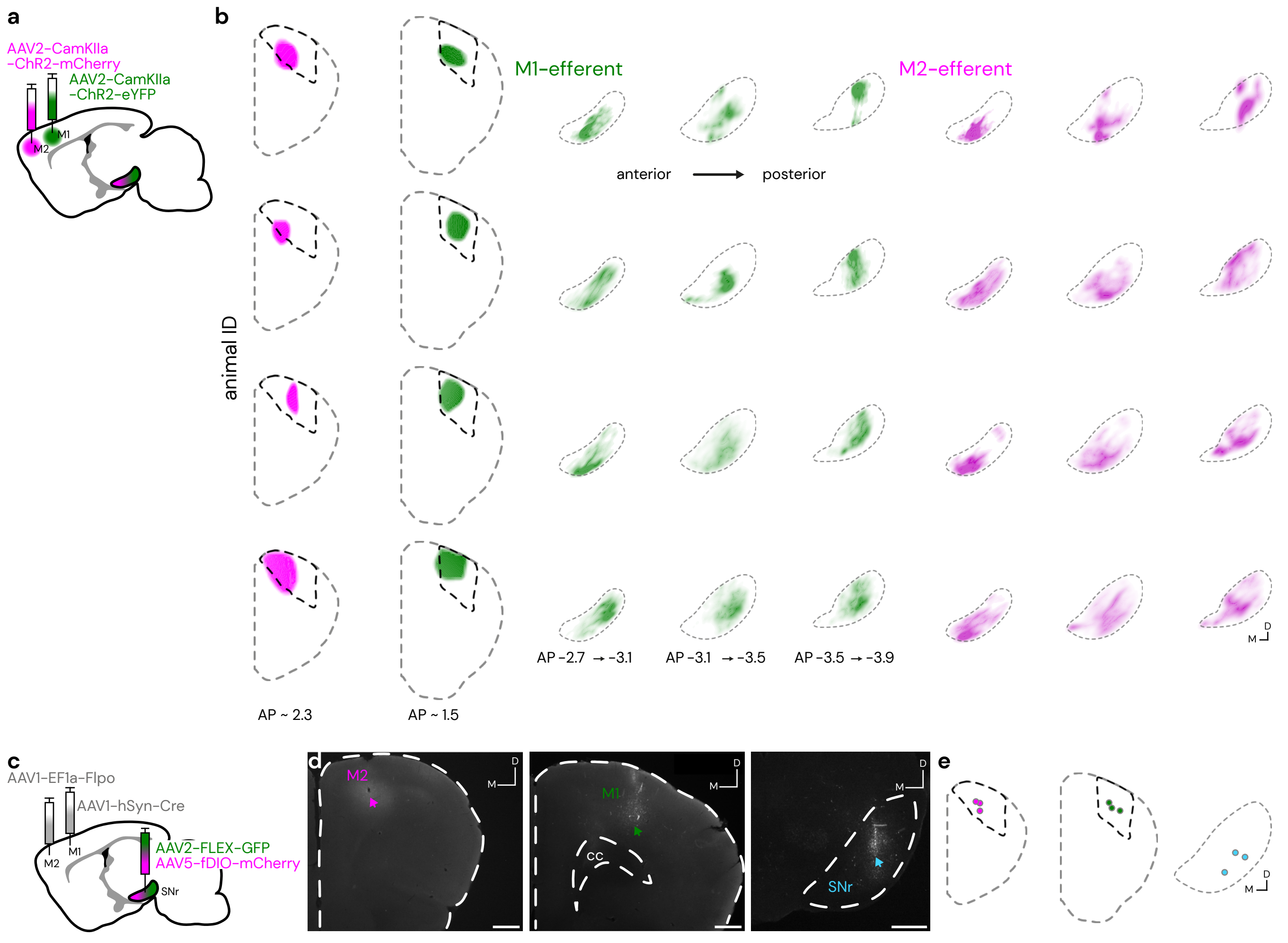
a
AAV2-CamKIIa
-ChR2-mCherry
AAV2-CamKIIa
-ChR2-eYFP
M1
M2
b
M1-efferent
M2-efferent
anterior
posterior
animal ID
AP -2.7 → -3.1
AP -3.1 → -3.5
AP -3.5 → -3.9
AP ~ 2.3
AP ~ 1.5
D
M
c
AAV1-EF1a-Flpo
AAV1-hSyn-Cre
AAV2-FLEX-GFP
AAV5-fDIO-mCherry
M2
M1
SNr
d
M2
M1
cc
SNr
D
M
e

Supplementary Fig. 6: Injection sites for anterograde axon tracing and anterograde transsynaptic labelling

a. Experimental setup. Simultaneous expression of mCherry and eYFP was driven in projection neurons of M1 and M2 via injection of viral vectors (AAV2-CamkIIa-ChR2-mCherry/AAV2-CamkIIa-ChR2-eYFP). **b.** Cortical injection sites (left M2, magenta; right: M1: green) and resulting axonal density across the anterior-posterior axis of the SNr for individual animals (N = 4), registered to the Allen Brain Institute Common Coordinate Framework. Delineations of M1 (left) and M2 (right) are indicated by dashed black lines. Anterior-posterior coordinates are indicated per column, in millimeters from Bregma. **c.** Experimental setup. Simultaneous expression of either mCherry or GFP was driven in SNr neurons receiving monosynaptic input from either M1 or M2 via injection of viral vectors (AAV1-hsyn-cre/AAV1-EF1a-flpo/AAV2-FLEX-GFP/AAV5-fDIO-mCherry). **d.** Example pipette tracts visible through autofluorescence in coronal brain slices. Maximum extent indicated by arrowheads (left M2, magenta; center: M1: green; right: SNr, white). **e.** Injection sites estimated from pipette tracts (left M2, magenta; center: M1, green, right: SNr, white) for all animals (N = 3). Corpus callosum indicated by cc in **d**. Scale bars: 500 µm.

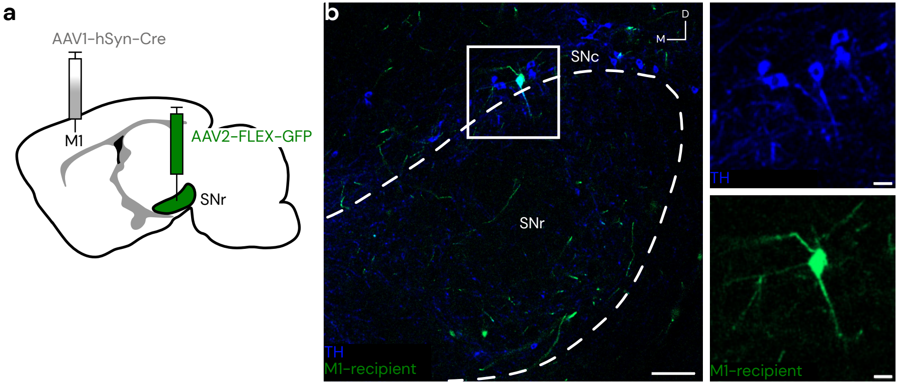

a
AAV1-hSyn-Cre
M1
AAV2-FLEX-GFP
SNr
b
D
M
SNc
SNr
TH
M1-recipient
TH
M1-recipient

Supplementary Fig. 7: Anatomical evidence for cortico-dopaminergic transmission

a. Experimental setup. Expression of GFP was driven in SNr neurons receiving monosynaptic input from M1 or M2 via injection of viral vectors (AAV1-hsyn-cre/AAV2-FLEX-GFP). **b.** A transsynaptically-labelled neuron in the substantia nigra pars compacta. Cell type was confirmed via tyrosine hydroxylase immunoreactiviy (TH, blue). Scale bars: 200 μm.

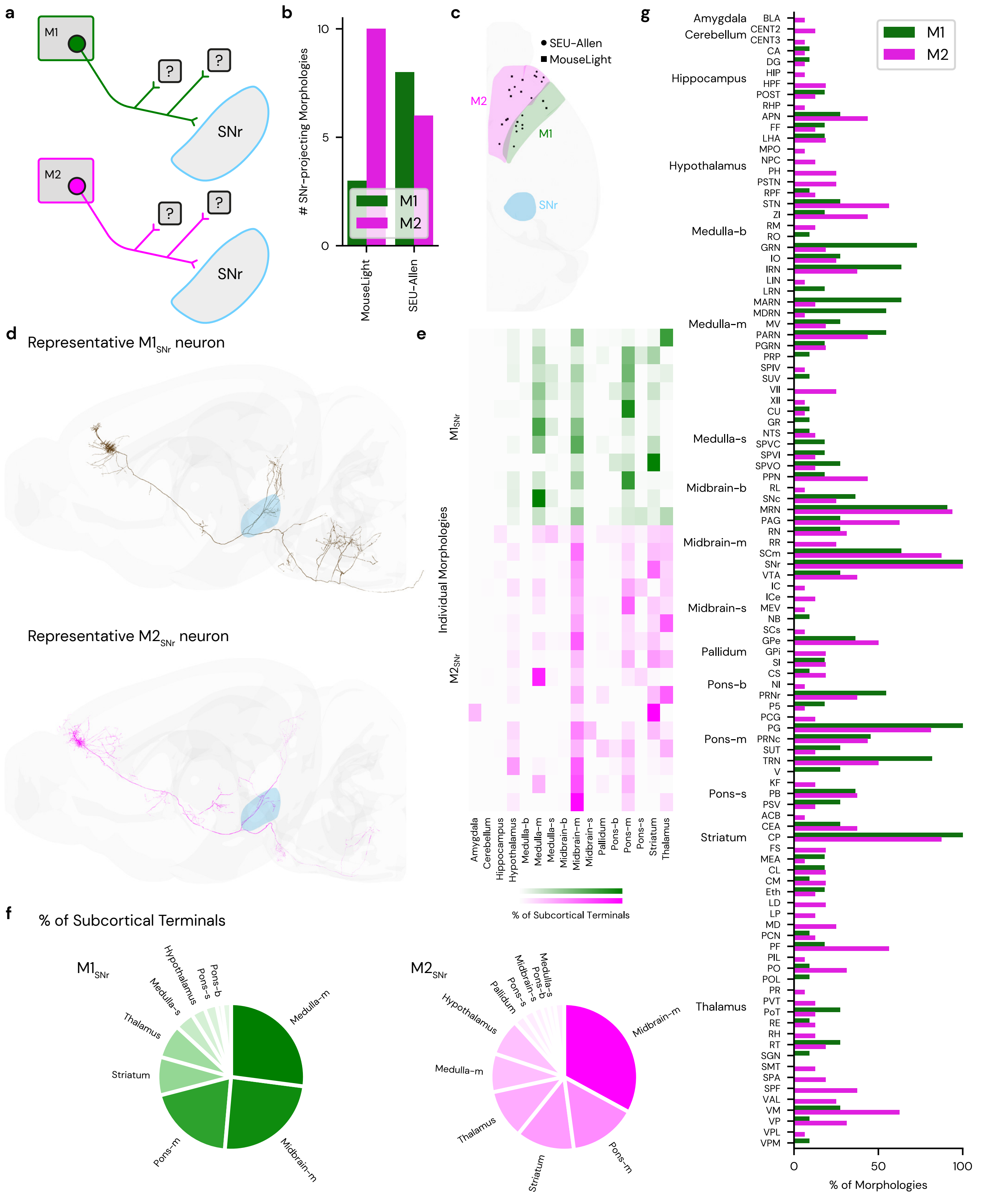
a
M1
M2
?
SNr
b
SNr-projecting Morphologies
M1
M2
MouseLight
SEU-Allen
c
SEU-Allen
MouseLight
M2
M1
SNr
d
Representative M1SNr neuron
Representative M2SNr neuron
e
M1SNr
M2SNr
Individual Morphologies
Amygdala
Cerebellum
Hippocampus
Hypothalamus
Medulla-b
Medulla-m
Medulla-s
Midbrain-b
Midbrain-m
Midbrain-s
Pallidum
Pons-b
Pons-m
Pons-s
Striatum
Thalamus
% of Subcortical Terminals
f
% of Subcortical Terminals
M1SNr
M2SNr
g
Amygdala
Cerebellum
Hippocampus
Hypothalamus
Medulla-b
Medulla-m
Medulla-s
Midbrain-b
Midbrain-m
Midbrain-s
Pallidum
Pons-b
Pons-m
Pons-s
Striatum
Thalamus
BLA
CENT2
CENT3
CA
DG
HIP
HPF
POST
RHP
APN
FF
LHA
MPO
NPC
PH
PSTN
RPF
STN
ZI
RM
RO
GRN
IO
IRN
LIN
LRN
MARN
MDRN
MV
PARN
PGRN
PRP
SPIV
SUV
VII
XII
CU
GR
NTS
SPVC
SPVI
SPVO
PPN
RL
SNc
MRN
PAG
RN
RR
SCm
SNr
VTA
IC
ICe
MEV
NB
SCs
GPe
GPi
SI
CS
NI
PRNr
P5
PCG
PG
PRNc
SUT
TRN
V
KF
PB
PSV
ACB
CEA
CP
FS
MEA
CL
CM
Eth
LD
LP
MD
PCN
PF
PIL
PO
POL
PR
PVT
PoT
RE
RH
RT
SGN
SMT
SPA
SPF
VAL
VM
VP
VPL
VPM
% of Morphologies

Supplementary Fig. 8: Analysis of SNr-targeting M1/M2 axonal reconstructions

a: Schematic of cortical collaterals. **b**: SNr-projecting morphologies found by querying SEU-Allen and MouseLight databases for neurons originating in M1 (green) or M2 (magenta). **c**: Horizontal view of the mouse brain, with M1 (green) and M2 (magenta) regions color-coded. Soma locations indicated by circles (SEU-Allen) or squares (MouseLight). **d**: Sagittal views of representative SNr-projecting neurons in M1 ($M1_{SNr}$; upper) or M2 ($M2_{SNr}$; lower). SNr indicated in blue. **e**: Heatmap indicating the percentage of subcortical axon terminals innervating each target region (x-axis), for each morphology (y-axis). $M1_{SNr}$ morphologies follow a white-green color gradient; $M2_{SNr}$ morphologies follow a white-magenta color gradient. Midbrain, pontine, and medullar regions are each subdivided into behavioral state-related ('-b'), motor-related ('-m'), and sensory-related ('-s'), as per the Allen Brain Institute Common Coordinate Framework ontology. **f**: Pie charts summarizing subcortical terminal distribution by region (as in **e**) for all $M1_{SNr}$ (n = 11) and $M2_{SNr}$ (n = 16) neurons. **g**: Barplot indicating the percentage of SNr-projecting morphologies targeting distinct brain structures ($M1_{SNr:}$ green; n = 11 neurons; $M2_{SNr:}$ magenta; n = 16 neurons). Brain structures are arranged first by encompassing region, and then alphabetically by acronym. ACB, Nucleus accumbens; APN, Anterior pretectal nucleus; BLA, Basolateral amygdalar nucleus; CA, Ammon's horn; CEA, Central amygdalar nucleus; CENT2, Lobule II; CENT3, Lobule III; CL, Central lateral nucleus of the thalamus; CM, Central medial nucleus of the thalamus; CP, Caudoputamen; CS, Superior central nucleus raphe; CU, Cuneate nucleus; DG, Dentate gyrus; Eth, Ethmoid nucleus of the thalamus; FF, Fields of Forel; FS, Fundus of striatum; GPe, Globus pallidus, external segment; GPi, Globus pallidus, internal segment; GR, Gracile nucleus; GRN, Gigantocellular reticular nucleus; HIP, Hippocampal region; HPF, Hippocampal formation; IC, Inferior colliculus; ICe, Inferior colliculus, external nucleus; IO, Inferior olivary complex; IRN, Intermediate reticular nucleus; KF, Koelliker-Fuse subnucleus; LD, Lateral dorsal nucleus of thalamus; LHA, Lateral hypothalamic area; LIN, Linear nucleus of the medulla; LP, Lateral posterior nucleus of the thalamus; LRN, Lateral reticular nucleus; MARN, Magnocellular reticular nucleus; MD, Mediodorsal nucleus of thalamus; MDRN, Medullary reticular nucleus; MEA, Medial amygdalar nucleus; MEV, Midbrain trigeminal nucleus; MPO, Medial preoptic area; MRN, Midbrain reticular nucleus; MV, Medial vestibular nucleus; NB, Nucleus of the brachium of the inferior colliculus; NI, Nucleus incertus; NPC, Nucleus of the posterior commissure; NTS, Nucleus of the solitary tract; P5, Peritrigeminal zone; PAG, Periaqueductal gray; PARN, Parvicellular reticular nucleus; PB, Parabrachial nucleus; PCG, Pontine central gray; PCN, Paracentral nucleus; PF, Parafascicular nucleus; PG, Pontine gray; PGRN, Paragigantocellular reticular nucleus; PH, Posterior hypothalamic nucleus; PIL, Posterior intralaminar thalamic nucleus; PO, Posterior complex of the thalamus; POL, Posterior limiting nucleus of the thalamus; POST, Postsubiculum; PPN, Pedunculopontine nucleus; PR, Perireunensis nucleus; PRNc, Pontine reticular nucleus, caudal part; PRNr, Pontine reticular nucleus; PRP, Nucleus prepositus; PSTN, Parasubthalamic nucleus; PSV, Principal sensory nucleus of the

trigeminal; PVT, Paraventricular nucleus of the thalamus; PoT, Posterior triangular thalamic nucleus; RE, Nucleus of reuniens; RH, Rhomboid nucleus; RHP, Retrohippocampal region; RL, Rostral linear nucleus raphe; RM, Nucleus raphe magnus; RN, Red nucleus; RO, Nucleus raphe obscurus; RPF, Retroparafascicular nucleus; RR, Midbrain reticular nucleus, retrorubral area; RT, Reticular nucleus of the thalamus; SCm, Superior colliculus, motor related; SCs, Superior colliculus, sensory related; SGN, Suprageniculate nucleus; SI, Substantia innominata; SMT, Submedial nucleus of the thalamus; SNc, Substantia nigra, compact part; SNr, Substantia nigra, reticular part; SPA, Subparafascicular area; SPF, Subparafascicular nucleus; SPIV, Spinal vestibular nucleus; SPVC, Spinal nucleus of the trigeminal, caudal part; SPVI, Spinal nucleus of the trigeminal, interpolar part; SPVO, Spinal nucleus of the trigeminal, oral part; STN, Subthalamic nucleus; SUT, Supratrigeminal nucleus; SUV, Superior vestibular nucleus; TRN, Tegmental reticular nucleus; V, Motor nucleus of trigeminal; VAL, Ventral anterior-lateral complex of the thalamus; VII, Facial motor nucleus; VM, Ventral medial nucleus of the thalamus; VP, Ventral posterior complex of the thalamus; VPL, Ventral posterolateral nucleus of the thalamus; VPM, Ventral posteromedial nucleus of the thalamus; VTA, Ventral tegmental area; XII, Hypoglossal nucleus; ZI, Zona incerta.

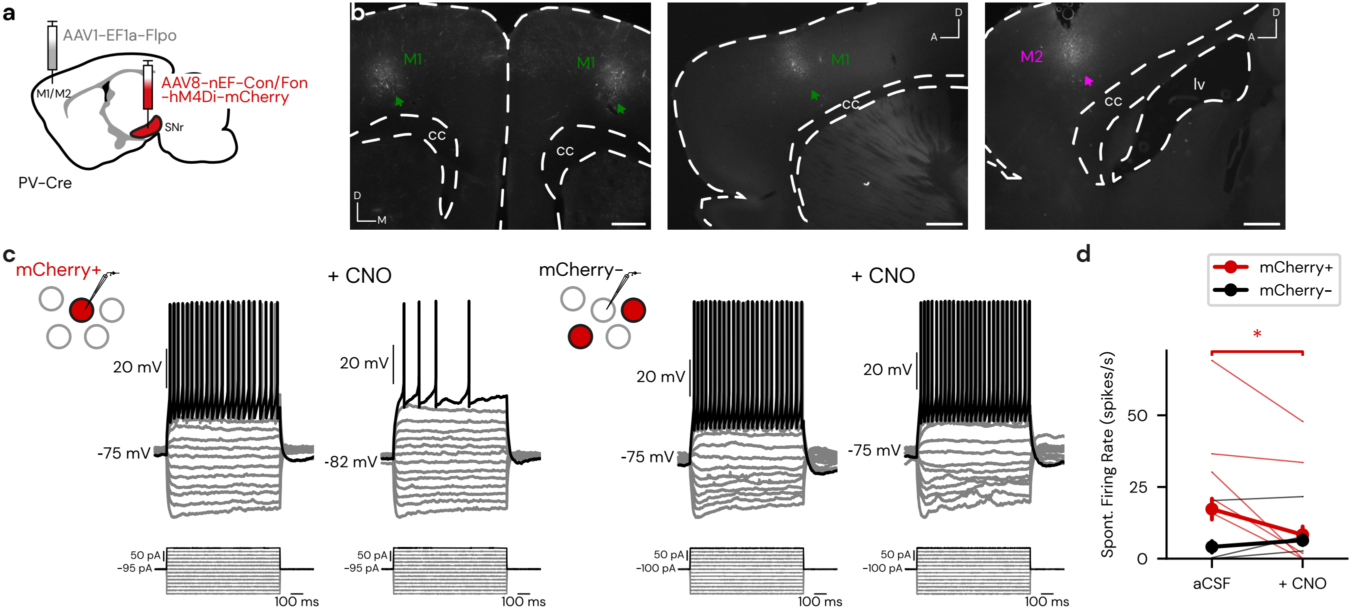

a
AAV1-EF1a-Flpo
M1/M2
AAV8-nEF-Con/Fon
-hM4Di-mCherry
SNr
PV-Cre
b
M1
M1
cc
cc
D
M
M1
cc
D
A
M2
cc
lv
D
A
c
mCherry+
20 mV
-75 mV
50 pA
-95 pA
100 ms
+ CNO
20 mV
-82 mV
50 pA
-95 pA
100 ms
mCherry-
20 mV
-75 mV
50 pA
-100 pA
100 ms
+ CNO
20 mV
-75 mV
50 pA
-100 pA
100 ms
d
mCherry+
mCherry-
*
Spont. Firing Rate (spikes/s)
0
25
50
aCSF
+ CNO

Supplementary Fig. 9: Validation of intersectional transsynaptic chemogenetic strategy

a. Experimental setup. AAV1-Ef1a-Flpo was injected bilaterally in either M1 or M2. AAV8-Con/Fon-hM4Di-mCherry was injected bilaterally in the SNr of PV-Cre mice. **b.** Example pipette tracts visible through autofluorescence in a coronal brain slice (left) and sagittal brain slices (center, right). Maximum extent indicated by arrowheads. **c.** Representative ex vivo membrane potential traces for mCherry-positive (left) and mCherry-negative (right) SNr neurons in aCSF, and following bath application of CNO. **d**. Summary of spontaneous firing rate changes (mCherry-positive: n = 5, N = 4 animals; mCherry-negative: n = 5 neurons, N = 4 animals; data pooled between M1- and M2-injected animals; * $p < 0.05$; two-tailed paired t-test). Data presented as mean ± SEM. Corpus callosum and lateral ventricle indicated by cc and lv, respectively, in **b**. Scale bars: 500 µm.

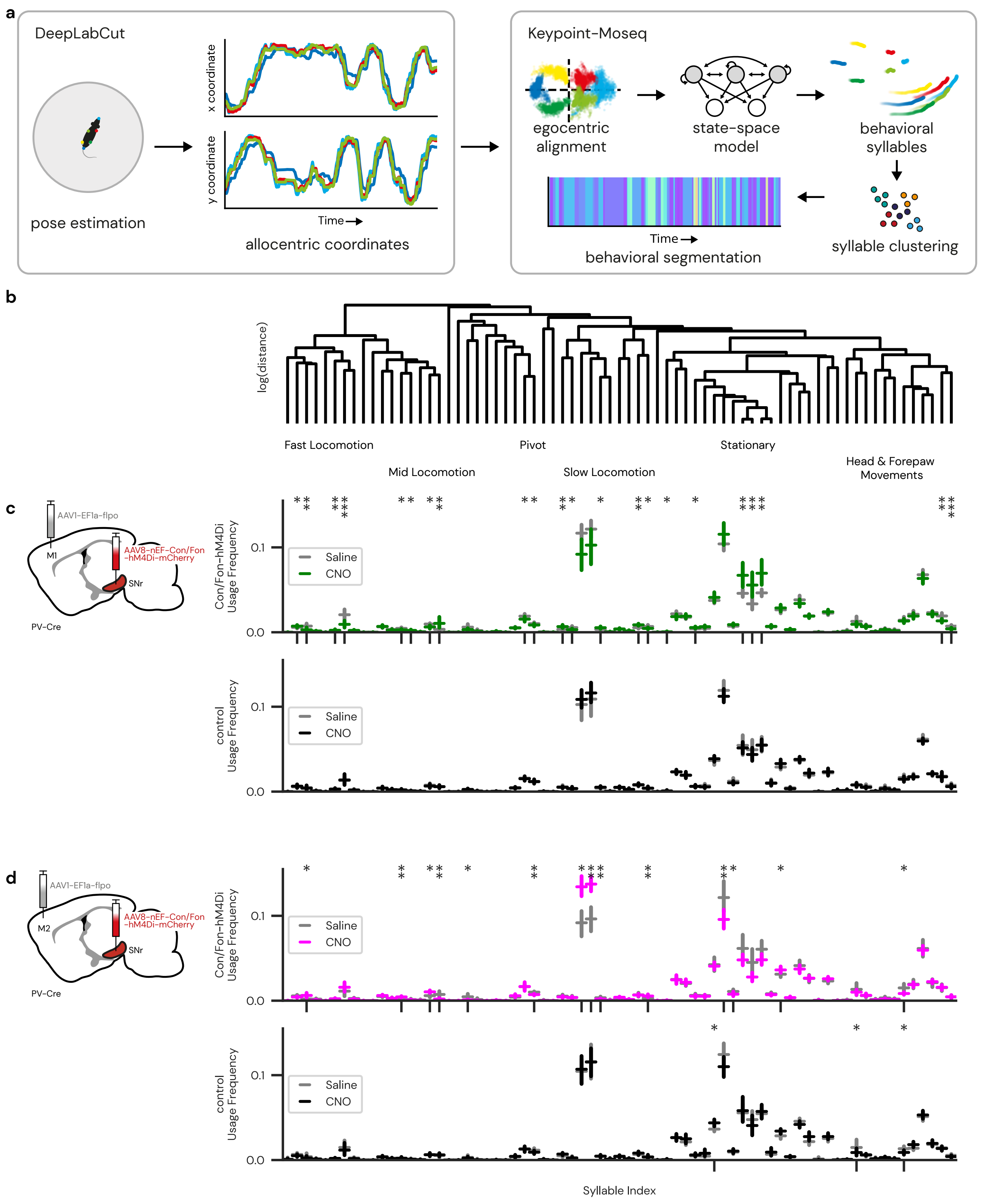
a
DeepLabCut
pose estimation
x coordinate
y coordinate
Time
allocentric coordinates
Keypoint-Moseq
egocentric alignment
state-space model
behavioral syllables
syllable clustering
Time
behavioral segmentation
b
log(distance)
Fast Locomotion
Mid Locomotion
Pivot
Slow Locomotion
Stationary
Head & Forepaw Movements
c
AAV1-EF1a-flpo
M1
AAV8-nEF-Con/Fon -hM4Di-mCherry
SNr
PV-Cre
Con/Fon-hM4Di Usage Frequency
control Usage Frequency
Saline
CNO
d
AAV1-EF1a-flpo
M2
AAV8-nEF-Con/Fon -hM4Di-mCherry
SNr
PV-Cre
Syllable Index

Supplementary Fig. 10: Segmentation of spontaneous behavior

a. Analysis overview. Allocentric coordinates obtained from DeepLabCut pose estimation are fed to Keypoint-Moseq[91]. Coordinates are converted to an egocentric reference frame and used to train a state-space model. Behavioral syllables are identified and clustered. Open field sessions are analyzed through the lens of transitions between behavioral syllables. **b:** Dendrogram of identified behavioral syllables with corresponding semantic labels. **c:** Syllable usage frequency for M1 Con/Fon-hM4Di (upper, N = 13) and control (lower, N = 12) animals. **d:** Same as **c**, for M2 Con/Fon-hM4Di (upper, N = 8) and control (lower, N = 11) animals (* $p < 0.05$, ** $p < 0.01$, *** $p < 0.001$; Wilcoxon signed rank test). Data presented as mean ± SEM.
