# Supplementary material for: Motor cortex directly excites the substantia nigra pars reticulata, the basal ganglia output nucleus
Source: Nat Commun. 2026 Jun 23;17:5551. doi: 10.1038/s41467-026-74569-w (PMC13291323; doi:10.1038/s41467-026-74569-w)
Supplement: Supplementary file 2 — Description of Additional Supplementary files [file 41467_2026_74569_MOESM2_ESM.pdf]

File name: Supplementary Movie 1

Description: Preserved locomotor capacity upon chemogenetic silencing of M1-recipient PV+ SNr neurons

A video showing a single locomotor bout of a mouse in a 35 cm diameter open field arena, recorded from below at 50 frames per second (FPS). The video features a PV+, M1-con/fon-hM4Di mouse, following intraperitoneal administration of Clozapine-n-oxide. DeepLabCut tracking of nose (blue) and tail base (green) points are overlaid over the video recording, with trails. The overlay in the top right corner indicates the direction and velocity of the mouse in polar coordinates (labelled: 'heading'). The overlay in the bottom left corner shows parallel (normalised) velocities of the nose point (blue) and tail base (green) in a rolling window. The current frame is indicated by a vertical line. The overlay in the bottom right corner shows the egocentric coordinates of the nose (blue), and both forepaws, in relation to the tail base (red cross). Playback is set to 50 FPS.

File name: Supplementary Movie 2

Description: Stationary behavior upon chemogenetic silencing of M1-recipient PV+ SNr neurons

A video showing a period of immobility of a mouse in a 35 cm diameter open field arena, recorded from below at 50 frames per second (FPS). The video features a PV+, M1-con/fon-hM4Di mouse, following intraperitoneal administration of Clozapine-n-oxide. DeepLabCut tracking of nose (blue) and tail base (green) points are overlaid over the video recording, with trails. The overlay in the top right corner indicates the direction and velocity of the mouse in polar coordinates (labelled: 'heading'). The overlay in the bottom left corner shows parallel (normalised) velocities of the nose point (blue) and tail base (green) in a rolling window. The current frame is indicated by a vertical line. The overlay in the bottom right corner shows the egocentric coordinates of the nose (blue), and both forepaws, in relation to the tail base (red cross). Playback is set to 50 FPS.

File name: Supplementary Movie 3

Description: Stationary behavior without head movements

A video showing a cut sequence of stationary behavior of a mouse in a 35 cm diameter open field arena, recorded from below at 50 frames per second (FPS).

The video features a PV+, M1-con/fon-hM4Di mouse, following intraperitoneal administration of Clozapine-n-oxide. DeepLabCut tracking of nose (blue) and tail base (green) points are overlaid over the video recording, with trails. The overlay in the top right corner indicates the direction and velocity of the mouse in polar coordinates (labelled: 'heading'). The overlay in the bottom left corner shows parallel (normalised) velocities of the nose point (blue) and tail base (green) in a rolling window. The current frame is indicated by a vertical line. The overlay in the bottom right corner shows the egocentric coordinates of the nose (blue), and both forepaws, in relation to the tail base (red cross). Playback is set to 50 FPS.

File name: Supplementary Movie 4

Description: Stationary behavior with small head movements

A video showing a cut sequence of stationary behavior, with small head movements, of a mouse in a 35 cm diameter open field arena, recorded from below at 50 frames per second (FPS). The video features a PV+, M1-con/fon-hM4Di mouse, following intraperitoneal administration of Clozapine-n-oxide. DeepLabCut tracking of nose (blue) and tail base (green) points are overlaid over the video recording, with trails. The overlay in the top right corner indicates the direction and velocity of the mouse in polar coordinates (labelled: 'heading'). The overlay in the bottom left corner shows parallel (normalised) velocities of the nose point (blue) and tail base (green) in a rolling window. The current frame is indicated by a vertical line. The overlay in the bottom right corner shows the egocentric coordinates of the nose (blue), and both forepaws, in relation to the tail base (red cross). Playback is set to 50 FPS.

File name: Supplementary Movie 5

Description: Locomotor behavior during chemogenetic silencing of M2-recipient PV+ SNr neurons

A video showing a single bout of locomotor behavior of a mouse in a 35 cm diameter open field arena, recorded from below at 50 frames per second (FPS). The video features a PV+, M2-con/fon-hM4Di mouse, following intraperitoneal administration of Clozapine-n-oxide. DeepLabCut tracking of nose (blue) and tail base (green) points are overlaid over the video recording, with trails. The overlay in the top right corner indicates the direction and velocity of the mouse in polar coordinates (labelled: 'heading'). The overlay in the bottom left corner shows

parallel (normalised) velocities of the nose point (blue) and tail base (green) in a rolling window. The current frame is indicated by a vertical line. The overlay in the bottom right corner shows the egocentric coordinates of the nose (blue), and both forepaws, in relation to the tail base (red cross). Playback is set to 50 FPS.

File name: Supplementary Movie 6

Description: Stationary behavior during chemogenetic silencing of M2-recipient PV+ SNr neurons

A video showing a single period of stationary behavior of a mouse in a 35 cm diameter open field arena, recorded from below at 50 frames per second (FPS). The video features a PV+, M2-con/fon-hM4Di mouse, following intraperitoneal administration of Clozapine-n-oxide. DeepLabCut tracking of nose (blue) and tail base (green) points are overlaid over the video recording, with trails. The overlay in the top right corner indicates the direction and velocity of the mouse in polar coordinates (labelled: 'heading'). The overlay in the bottom left corner shows parallel (normalised) velocities of the nose point (blue) and tail base (green) in a rolling window. The current frame is indicated by a vertical line. The overlay in the bottom right corner shows the egocentric coordinates of the nose (blue), and both forepaws, in relation to the tail base (red cross). Playback is set to 50 FPS.
