# Supplementary material for: Motor cortex directly excites the substantia nigra pars reticulata, the basal ganglia output nucleus
Source: Nat Commun. 2026 Jun 23;17:5551. doi: 10.1038/s41467-026-74569-w (PMC13291323; doi:10.1038/s41467-026-74569-w)
Supplement: Supplementary file 9 — Reporting Summary [file 41467_2026_74569_MOESM9_ESM.pdf]

nature portfolio

Corresponding author(s): GIlad Silberberg
Sten Grillner

Last updated by author(s): Jun 2, 2026

Reporting Summary

Nature Portfolio wishes to improve the reproducibility of the work that we publish. This form provides structure for consistency and transparency in reporting. For further information on Nature Portfolio policies, see our Editorial Policies and the Editorial Policy Checklist.

Statistics

For all statistical analyses, confirm that the following items are present in the figure legend, table legend, main text, or Methods section.

n/a	Confirmed	
☐	☒	The exact sample size (*n*) for each experimental group/condition, given as a discrete number and unit of measurement
☐	☒	A statement on whether measurements were taken from distinct samples or whether the same sample was measured repeatedly
☐	☒	The statistical test(s) used AND whether they are one- or two-sided<br>*Only common tests should be described solely by name; describe more complex techniques in the Methods section.*
☒	☐	A description of all covariates tested
☐	☒	A description of any assumptions or corrections, such as tests of normality and adjustment for multiple comparisons
☐	☒	A full description of the statistical parameters including central tendency (e.g. means) or other basic estimates (e.g. regression coefficient) AND variation (e.g. standard deviation) or associated estimates of uncertainty (e.g. confidence intervals)
☐	☒	For null hypothesis testing, the test statistic (e.g. *F*, *t*, *r*) with confidence intervals, effect sizes, degrees of freedom and *P* value noted<br>*Give P values as exact values whenever suitable.*
☒	☐	For Bayesian analysis, information on the choice of priors and Markov chain Monte Carlo settings
☒	☐	For hierarchical and complex designs, identification of the appropriate level for tests and full reporting of outcomes
☒	☐	Estimates of effect sizes (e.g. Cohen's *d*, Pearson's *r*), indicating how they were calculated

Our web collection on statistics for biologists contains articles on many of the points above.

Software and code

Policy information about availability of computer code

Data collection	For electrophysiological experiments, Igor Pro (version 6.37, Wavemetrics) was used for acquisition. For imaging experiments Zen Blue (version 2.1, Zeiss) was used for acquisition. For behavioural experiments, Spinnaker SDK (version 4.2.0.83, Teledyne FLIR) was used to acquire video.
Data analysis	For electrophysiological experiments, open source python packages: eFEL (version 5.6.26), Elephant (version 1.2), NEO (0.14.0), and scipy (version 1.15.2) were used for analysis. For imaging experiments, the open source python package DMC-Brainmap (version 0.1.7) was used for analysis. The open source processing package FIJI (version 2.16.0) was used to adjust brightness and contrast, and to convert file formats. Morphological reconstructions were generated with open source software neuTube (version 0.9.18). The open source computer graphics software Blender (version 4.0) was used for 3D visualisations. For behavioural experiments, videos were analysed using DeepLabCut (version 3.0). Statistical analysis of behavioural data was performed with the open source python package scipy (version 1.15.2). Further analysis of behavioural data was performed with the open source python package Keypoint-MoSeq (version 0.6.7).

For manuscripts utilizing custom algorithms or software that are central to the research but not yet described in published literature, software must be made available to editors and reviewers. We strongly encourage code deposition in a community repository (e.g. GitHub). See the Nature Portfolio guidelines for submitting code & software for further information.

Data

Policy information about availability of data

All manuscripts must include a data availability statement. This statement should provide the following information, where applicable:

- Accession codes, unique identifiers, or web links for publicly available datasets
- A description of any restrictions on data availability
- For clinical datasets or third party data, please ensure that the statement adheres to our policy

A source data file supporting the findings of this study is provided with this paper. Further data are available from the authors upon request.

Research involving human participants, their data, or biological material

Policy information about studies with human participants or human data. See also policy information about sex, gender (identity/presentation), and sexual orientation and race, ethnicity and racism.

Reporting on sex and gender	*Use the terms sex (biological attribute) and gender (shaped by social and cultural circumstances) carefully in order to avoid confusing both terms. Indicate if findings apply to only one sex or gender; describe whether sex and gender were considered in study design; whether sex and/or gender was determined based on self-reporting or assigned and methods used. Provide in the source data disaggregated sex and gender data, where this information has been collected, and if consent has been obtained for sharing of individual-level data; provide overall numbers in this Reporting Summary. Please state if this information has not been collected. Report sex- and gender-based analyses where performed, justify reasons for lack of sex- and gender-based analysis.*
Reporting on race, ethnicity, or other socially relevant groupings	*Please specify the socially constructed or socially relevant categorization variable(s) used in your manuscript and explain why they were used. Please note that such variables should not be used as proxies for other socially constructed/relevant variables (for example, race or ethnicity should not be used as a proxy for socioeconomic status). Provide clear definitions of the relevant terms used, how they were provided (by the participants/respondents, the researchers, or third parties), and the method(s) used to classify people into the different categories (e.g. self-report, census or administrative data, social media data, etc.) Please provide details about how you controlled for confounding variables in your analyses.*
Population characteristics	*Describe the covariate-relevant population characteristics of the human research participants (e.g. age, genotypic information, past and current diagnosis and treatment categories). If you filled out the behavioural & social sciences study design questions and have nothing to add here, write "See above."*
Recruitment	*Describe how participants were recruited. Outline any potential self-selection bias or other biases that may be present and how these are likely to impact results.*
Ethics oversight	*Identify the organization(s) that approved the study protocol.*

Note that full information on the approval of the study protocol must also be provided in the manuscript.

Field-specific reporting

Please select the one below that is the best fit for your research. If you are not sure, read the appropriate sections before making your selection.

☒ Life sciences ☐ Behavioural & social sciences ☐ Ecological, evolutionary & environmental sciences

For a reference copy of the document with all sections, see nature.com/documents/nr-reporting-summary-flat.pdf

Life sciences study design

All studies must disclose on these points even when the disclosure is negative.

Sample size	No a priori sample size calculation was performed. For electrophysiological recordings, we sought to record from a total number of neurons on par with previous publications studying the distribution of electrophysiological properties or SNr neuron subpopulations (e.g., McElvain et al., 2021; 10.1016/j.neuron.2021.03.017). For anatomical tracing, at least 3 animals were used in each experiment. For behavioural experiments, at least 8 animals were included in each group. These values were chosen based on prior studies performing similar analyses (e.g., Yang et al., 2023; 10.1016/j.cell.2022.12.009).
Data exclusions	For electrophysiological recordings, animals were included if viral expression was visible around the cortical injection site (100% of cases). Individual cells were excluded for analysis if the patch clamp seal deteriorated before a) a complete electrophysiological profile and b) testing of optogenetic stimulation could be run.<br><br>For anatomical tracing, no animals were excluded from analysis. Individual cells were not counted if they colocalised with tyrosine hydroxylase immunoreactivity.

	Individual animals were excluded from behavioral analysis following post hoc confirmation that viral expression was a) present, and b) limited to the region of interest (SNr).
Replication	All main results were reproduced in multiple animals, and multiple neurons with exact n numbers for each experiment provided in the manuscript.
Randomization	Mice were selected on availability up to 3 months of age at time of initial procedure, which produced an even distribution of sex and age. For behavioural experiments, PV-cre mice were randomly assigned M1 or M2 AAV1-hSyn-cre injections.
Blinding	For electrophysiological recordings, patching was performed blindly in the slice (i.e., no cell type identifiers), with the caveat that fluorescently-labelled afferent fibers were visible.<br><br>For anatomical tracing, blinding was not possible, but all analysis was automated.<br><br>For behavioural experiments, the experimenter was blinded to injection site and genotype. Regardless, procedures were standardized to remove possible subjective manipulation.

Reporting for specific materials, systems and methods

We require information from authors about some types of materials, experimental systems and methods used in many studies. Here, indicate whether each material, system or method listed is relevant to your study. If you are not sure if a list item applies to your research, read the appropriate section before selecting a response.

Materials & experimental systems

n/a	Involved in the study
☐	☒ Antibodies
☒	☐ Eukaryotic cell lines
☒	☐ Palaeontology and archaeology
☐	☒ Animals and other organisms
☒	☐ Clinical data
☒	☐ Dual use research of concern
☒	☐ Plants

Methods

n/a	Involved in the study
☒	☐ ChIP-seq
☒	☐ Flow cytometry
☒	☐ MRI-based neuroimaging

Antibodies

Antibodies used	rabbit anti-tyrosine hydroxylase polyclonal antibody (Sigma-Aldrich, U.S.A.; Cat: AB152)<br>rabbit anti-mCherry polyclonal antibody (Abcam, U.K.; Cat: ab167453)<br>Cy3-conjugated goat, or Cy5-conjugated donkey, anti-rabbit polyclonal secondary antibody (The Jackson Laboratory, U.S.A.; Cat: 111-165-003 and 705-165-147)
Validation	Anti-tyrosine hydroxylase antibodies were used as described in previous published works (e.g., 10.1016/j.neuron.2017.05.004, 10.1523/JNEUROSCI.0852-07.2007). Anti-mCherry antibody was used as per manufacturers instructions. Per the manufacturer, this antibody has been used in 471 publications since first being made available in 2014.

Animals and other research organisms

Policy information about studies involving animals; ARRIVE guidelines recommended for reporting animal research, and Sex and Gender in Research

Laboratory animals	wild type mice (C57BL/6J, stock #000664, The Jackson Laboratory, U.S.A.), and heterozygous PV-Cre mice (stock #000664, The Jackson Laboratory, U.S.A.; maintained on a C57BL/6J background) were used in this study.
Wild animals	This study did not involve wild animals.
Reporting on sex	Both male and female mice were used in this study. Sex differences were not analyzed due to limited sample sizes.
Field-collected samples	This study did not involve samples collected from the field
Ethics oversight	Ethical approval was provided by the local ethical board, Stockholm Norra Djurförsöksetiska Nämnd, under an ethical permit to Gilad Silberberg (N2022-2020).

Note that full information on the approval of the study protocol must also be provided in the manuscript.

Plants

Seed stocks	N/a
Novel plant genotypes	N/a
Authentication	N/a
