# Supplementary material for: Motor cortex directly excites the substantia nigra pars reticulata, the basal ganglia output nucleus
Source: Nat Commun. 2026 Jun 23;17:5551. doi: 10.1038/s41467-026-74569-w (PMC13291323; doi:10.1038/s41467-026-74569-w)
Supplement: Supplementary file 10 — Transparent Peer Review file [file 41467_2026_74569_MOESM10_ESM.pdf]

nature portfolio

Peer Review File

Motor cortex directly excites the substantia nigra pars reticulata, the basal ganglia output nucleus

Corresponding Author: Professor Gilad Silberberg

This file contains all reviewer reports in order by version, followed by all author rebuttals in order by version.

Version 0:

Reviewer comments:

Reviewer #1

(Remarks to the Author)
The study by Thompson and coworkers provides strong evidence for monosynaptic glutamatergic inputs to the substantia nigra pars reticulata from projection neurons in both the primary and secondary motor cortices. The anatomical connectivity and role of these inputs and the targeted neurons in spontaneous locomotion were also examined. The authors used an impressive combination of anatomical, physiological, chemogenetic, optogenetic and behavioral experiments to provide this new evidence of the function of a cortico-nigral "ultradirect" pathway. They also took advantage of existing imaging databases to chart the collateral projections of these cortical neurons. Overall, the manuscript investigates a relatively understudied yet important neural circuit component within the basal ganglia using an ambitious framework. Previous studies had only provided anatomical evidence for this connection, and thus the convincing electrophysiological evidence for the monosynaptic cortico-nigral synapses is valuable to the field. Given that the SNr integrates projection inputs from multiple brain regions, dissociating the specificity of its functional role or downstream targets is a significant challenge.
The study provides valuable new information that will force a re-evaluation of key aspects of basal ganglia function. The roles of these projections in spontaneous locomotion is an important aspect of the study. If the results are supported as currently presented, this suggests interesting contributions to action initiation or termination (but see major comment 1 below). The discussion includes an informative consideration of how cortico-nigral connections could contribute to basal ganglia function and movement control. The possibility of convergence with other afferents innervating SNr is an interesting topic nicely discusse by the authors. While we acknowledge the importance of this scientific question and the authors' efforts, we find that the data presented in the manuscript are insufficient to provide readers with a comprehensive understanding of the circuit in question. Some findings are overinterpreted given the limited supporting evidence. In particular there are concerns about the evidence for anatomical segregation of the M1 and M2 inputs to SNr, and the behavioral data lack sufficient quantification and completeness to clarify the specific role of these inputs in locomotion.

Major Comments

1. The caption for figure 6 appears to indicate that only 2-3 animals were examined in the chemo- and optogenetic experiments. Furthermore, the graphs in figure 6d,e,i and j do not indicate if there were significant differences across the groups (and the data only show mean and error bars with no points showing individual values in the different groups). Is this accurate? If these sample sizes are correct, then these experiments are woefully underpowered and the experiment needs to be repeated with reasonable samples sizes. Furthermore, it would be useful to show the individual values for each group so that the readers can appreciate the true range of values.

2. In addition, in figure 6d it appears that the GFP group shows greater distance traveled in the saline condition as compared to the other two groups. Could there be an effect of expression of the hM4D(Gi) construct itself, independent of agonist treatment?

3. Could additional analyses help to clarify the role of the cortical-SNr projections in spontaneous locomotion. For example, do animals move for shorter periods of time or simply not initiate as many movement bouts when the neurons or projections are inhibited? Deeper analysis of this behavior might help to reveal what aspects of movement are likely to involve the cortico-nigral projections.

4. The topographical segregation of M1 and M2 projections in the SNr is a central claim of the manuscript. However, the

authors only present limited data directly supporting this claim. For example, they do not show images or analysis of M2-SNr terminals from the experiments in which ChR2 was injected into M2, and the image in figure 6g appears to show only sparse M2 innervation around the SNr. Regarding the M1 projections, figure 1b appears to show relatively dense terminal labeling, but a lower magnification image is needed to show where this is within SNr. Likewise, the somatic labeling in figure 5b is not dense and does not appear to adequately represent the topographical features of the M1/M2 projections in the SNr as described in the text and figure 5d and e. The authors should show the images from all injected animals in the supplement for both the ChR2 and anterograde labeling experiments to provide more detailed information about innervation.

5. More information is also needed about the viral scheme and graphical approach used in Figures 5d and e. The manuscript must clearly detail the methods for data collection and processing. Furthermore, there is a significant discrepancy between the raw fluorescence image in Figure 5c and the processed data in Figure 5e. unbiased data analysis and statistical validation of Figure 5e are necessary to support the claim of spatial segregation.

6. A straightforward and effective way to demonstrate the segregation of projections would be through a two-color viral injection (e.g., GFP in M1 or mCherry in M2) to visualize the fluorescence distribution of afferents and terminals in the SNr. It would be beneficial to use FLP and CRE based schemes to image both SNr/M1 and SNr/M2 in the same brain, if possible. As it stands, the manuscript does not provide essential evidence to substantiate this key assertion.

7. Dopaminergic neurons could be directly activated by motor cortex projections (Whole-Brain Mapping of Direct Inputs to Midbrain Dopamine Neurons, M. Watabe-Uchida et.al , Neuron, 2012), although admittedly few such connections were observed in this study. However, these sparse connections could still contribute to the findings in Figures 6f-j. The fluorescence vacancy observed in Figure 6g, particularly in the SNr, raises further concerns. While the optogenetic manipulation of M1/M2 terminals is an interesting approach, this manipulation could potentially lead to activation of both SNr neurons and dopaminergic neurons. I believe expressing an inhibitory opsin in the SNr or specifically in SNr-projecting cortical neurons would be a valuable additional approach.

Minor Comments

i. Can the authors provide any information about the cortical layers that contain the somata of the cortico-nigral neurons? One would presume that these are deep layer neurons, but do they arise from layer V or VI?

ii. The examination of potential S1 cortex-SNr projections is a useful part of the story. It would be helpful to know if other cortical regions project directly to SNr, but admittedly this is beyond the scope of the present study. This possibility should be discussed however, and it would be helpful to know if the literature suggests that any other cortical neurons project directly to SNr. This would help readers to evaluate if this is a unique property of the motor cortices.

iii. Figure 6b, (DIO-hM4Di expression), if the authors applied the same cell analysis technique used in Figures 5d-h (DIO-eGPF) , would they reach the same conclusion?

iv. From the histology analysis, what percentage of SNr neurons are directly innervated by either M1 or M2? If this percentage is low how can they exert a dominant influence on locomotion while other functionally active neurons in the SNr are present?

v. Figure 6f, if the behavioral indicators are collected over the entire 300-second period (x4), is it possible to provide a more detailed time course of locomotion before and during the optogenetic light exposure, rather than just 3 time points?

vi. Figure 4a, the action potentials during the 20Hz stimulation are very tightly packed and some appear to have decreased amplitudes. Perhaps the authors should include an inset with an expanded timescale time axis so that individual spikes can be more readily distinguished.

vii. Page 14, the authors should indicate the GFP vector that was injected as the control for the chemogenetic experiments.

viii. Page 9, 1st line, the authors refer to the parvicellular reticular nucleus. Should this be parvocellular? Two lines later, perhaps “pontine” should be “pons”?

Reviewer #2

(Remarks to the Author)
The work by Thompson et al demonstrated a monosynaptic pathway from the motor cortex to the substantia nigra pars reticulata (SNr), which is the main basal ganglia output nuclei in rodents. This work is timely and important to the basal ganglia field because the direct cortico-nigral pathway provides a cortical access to the output structure of the basal ganglia by passing the traditional basal ganglia direct and indirect pathway models. The work is original, rigorously designed, and well conducted and will advance our understanding of cortico-basal ganglia circuits in regulating motor and nonmotor function. I only have a few minor comments that may help to further consolidate the conclusion of this study.
1. The exitence of cortico-nigral projection can be further strengthened by additional histology studies. For example, quantification of the presence of the vesicular glutamate transporter 1 (vGluT1) immunoreactivity in the SNr will complement with the existing electrophysiology data. It would be even informative to compare the relative density of vGlu1 puncta versus vGluT2 puncta in the SNr, providing an estimated relative innervation of SNr neurons by the cortical inputs versus

subthalamic inputs.
2. Does the globus pallidus internal part (GPi, another basal ganglia output nuclei in rodents) also receive monosynaptic inputs?
3. The existence of direct projection from the cortex to the basal ganglia output nuclei was indicated in the literature. The work by Milardi et al (2015) (PMID: 25156805) should be discussed.
4. The data in Figure 2 showing the lack of NMDARs-mediated glutamatergic transmission are surprising and unexpected. What does it mean in terms of synaptic plasticity and motor learning or general motor control? In the absence of postsynaptic NMDRs, do the cortico-nigral synapses functionally express Ca2+ permeable AMPARs? The subthalamus nucleus (STN) provides the major excitation to the SNr GABAergic neurons. Does the STN-SNr transmission involve NMDARs? Further experimental validation and clarification on NMDAR-mediated EPSC/EPSP may help to address potential technical issues during the recording.
5. It would be helpful to clarify how much overlap between cortico-nigral pathway and corticospinal/cortical-STN projections in rodents. See Milardi et al (2015) (PMID: 25156805) for additional information in human.

Reviewer #3

(Remarks to the Author)
I co-reviewed this manuscript with one of the reviewers who provided the listed reports. This is part of the Nature Communications initiative to facilitate training in peer review and to provide appropriate recognition for Early Career Researchers who co-review manuscripts.

Reviewer #4

(Remarks to the Author)
Thompson and colleagues conducted a compelling series of experiments demonstrating the cortico-nigral pathway. They used anatomical, electrophysiological, chemogenetic, and optogenetic techniques to elucidate this pathway both structurally and functionally. Their approach was thorough and their findings were convincing. This work adds a new and important pathway to the basal ganglia circuit, one likely to have behavioral and clinical relevance.

The majority of their experiments were fastidiously executed, addressing any concerns I might have had as I was reading through the Results. The one exception was the final pair of experiments, the behavioral studies.

1. The behavioral studies. In the abstract, they're a good pair of experiments: you modulate the presynaptic side of the pathway, you modulate the postsynaptic side of the pathway, and the results end up being consistent with each other. But the problem I see is that the SNc could have been affected in both experiments, and that could explain the results. Dopaminergic neurons of the SNc receive cortical input from M1 and M2 (Watabe-Uchida et al 2012 Neuron), dopaminergic neuron activity increases during movement (Schultz et al 1983 Exp Brain Res), and inhibition of SNc neurons is aversive (Ilango et al. 2014 J Neurosci), which seems like it could lead to akinesia during inhibition. And since the SNr and SNc are right next to each other, it's hard to affect one without affecting the other. Unless more is done to address this alternative, the cortico-SNr pathway can't be described as the sole mediator of the behavioral effects you recorded.

1a. In the DREADD experiment, did any dopaminergic neurons of the SNc express the receptor? When I looked carefully at Fig. 6b, I didn't see any, but were all sections free of receptor expression in the SNc? Were all receptor-expressing neurons TH negative?

1b. The optogenetic experiment may also be subject to the same issue of potentially affecting the SNc. In Fig 6g, the tip of the optic fiber appears to end right in the middle of a big bundle of opsin-expressing cortical axons that pass through the SNc. Can you offer evidence that only or mostly the SNr was affected in this experiment? In your trans-synaptic labeling experiments (from Fig. 5) did you see any labeling of SNc neurons?

1c. Also, were the DREADDs and opsins expressed unilaterally or bilaterally? I saw in Methods your given surgical coordinates were unilateral, so I assume your interventions were unilateral, but clearly stating that in the body of the paper would be helpful. Unilateral manipulations of the basal ganglia in movement studies have a tendency to make the animal rotate or walk in circles, rather than exhibit strict immobility (e.g., Chen, Chan & Yung 2002 Neuroscience), so your behavioral findings, if unilaterally generated, would stand out.

Minor points/questions.

1. What wattage of light was used in the electrophysiological recording experiments?

2. The 'widefield photostimulation' of M1 and M2 was aimed where: the whole slice, M1/M2, or SNr?

3. In Figure 2d the labels on the pie chart are unclearly placed.

4. In Fig. 5d the brain is a little hard to see.

5. In the section, “Photoactivation of motor cortex axon terminals increases SNr firing rate ex vivo,” were coronal or sagittal slices used (or both)? The Methods for the recording experiments states that slices were either coronal or sagittal. In sagittal slices it is possible that the whole cortico-striatal-nigral pathway is intact, and stimulation of channelrhodopsin-expressing terminals in the nigra could antidromically activate cortical axons throughout the whole descending pathway, indirectly stimulating other components of the basal ganglia like the STN. Also clarify whether drugs were added to the solutions to record monosynaptic activity only. Again, I know the Methods describes that such drugs were used at times, but adding it to the Results section makes it unequivocal, and I don’t have to go digging around in the Methods section for that detail.

Reviewer #5

(Remarks to the Author)
This manuscript by Thompson et al. sets out to test whether the previously described, but hardly explored projections from motor cortex (primary M1 and secondary M2) to the substantia nigra pars reticulata (SNr) are functional, whether projections from M1 and M2 target distinct SNr neuron populations and whether their manipulation has behavioral effects. For this, they optogenetically activate axon terminals of M1 or M2 neurons in SNr while patching SNr neurons. They found that about half of the neurons receive monosynaptic AMPA-mediated inputs. They further show that M1- and M2-targeted SNr neurons have some distinct properties (firing threshold, spontaneous firing rate). Furthermore, they show that the motor cortex to SNr synapses have mixed properties, some showing facilitation, some depression in the paired pulse ratio. They also show in cell-attached ex vivo recordings that motor cortex inputs can modulate the firing of targeted SNr neurons. Anatomical tracing using anterograde AAV1s shows that M1- or M2-targeted SNr neurons have distinct projection patterns to other subcortical areas. Taken together with their patch results, this suggests that M1 and M2 target distinct populations of SNr neurons. Finally, the authors show experiments suggesting that the M1/M2 to SNr projections can affect spontaneous behavior – silencing of M1 or M2 axon terminals in SNr or of targeted SNr neurons, using either opto- or chemogenetics, reduces the distance travelled in an open field test.
The characterization of the previously described, but largely unexplored projections from motor cortex directly to the SNr is important and of high interest, since these projections add another control pathway via which cortex may directly influence motor behavior – either in coordination with or bypassing the upstream nuclei of the basal ganglia. The combination of careful in vitro characterization of the connectivity, anatomical tracing and behavior is powerful. Especially the in vitro patch clamp experiments are rigorously done. However, an incomplete description/ characterization of their injection sites and tracing as well as very preliminary behavioral results dampen the enthusiasm. Below are my individual concerns:

Major:
- Behavior: The animal numbers for the behavior are too low to draw any reliable conclusions. They have groups of only 2-3 animals for both their chemo- and optogenetic experiments. While the results show the trends they are describing, they are highly variable (even at baseline) and no statistics have been performed (also not meaningfully possible with such low n). Currently, the behavior results are not convincing, and additional animals need to be added.
- Behavior: They seem to only inhibit chemo- or optogenetically unilaterally. While bilateral inhibition would have likely provided clearer results, they should at the very least test whether these unilateral manipulations lead to any lateralized effects on behavior, e.g., turning.
- Behavior: The analysis is rather superficial. While I understand that they try to just show that behavior is affected at all, it would be good if they could make more use of their data. In addition to looking at, e.g., turning (see above), they could also look at measurements like running bout number and length (indication for effects on action initiation), changes in center vs. wall locations etc.
- Behavior: they did not validate the PdCO approach – since axon terminal inhibition comes with caveats, it would have been good to see, at least in a slice, that their approach indeed does what it is supposed to do. I am aware that this tool has been published, but it has not been widely used, and its effects may vary across different types of projections
- Histology: They need to show/quantify their injection sites for all experiments. They make claims about specific targeting in SNr etc., but the observed patterns might be due to their specific injection sites. Controlling for this is especially important given that they claim a clear topography of M1/M2 inputs in SNr.
- Histology: They do not specify their inclusion/exclusion criteria for any of their experiments – were animals with too big/ too small injections excluded? Did they quantify injection sites to determine exclusion?
- Tracing: Fig. 5 – their axon tracing for SNr neurons that are targeted by M1 or M2 seems preliminary. There are no quantifications, no comparisons across animals, etc. While the shown examples are intriguing, this seems insufficient. Especially combined with the lack of a presentation and quantification of the injection sites. Are the differences in M1 and M2 experiments maybe due to incomplete/variable injections?
- Tracing: Fig. 5 - they trace the axons of SNr neurons that are labeled using AAV1-Cre and AAV2-FLEX-GFP. It is important to confirm that there are no neurons labeled outside SNr – otherwise, the axons they trace in other areas might originate from such non-SNr neurons. From the one example image (Fig. 5c) it is hard to see, but already there may potentially be labeled neurons outside SNr.
- Tracing Fig. 5: related to above – does the AAV2-FLEX injection cover the same area in SNr across M1/M2 animals – otherwise, any difference may be misleading

Minor:
- ED Fig. 1: they state in the legend they have recorded from 38 neurons in 51 animals. They should state the actual number of animals from which these neurons are recorded.
- Fig. 6a: Why does the scheme mention ‘ex vivo electrophysiology’?

Version 1:

Reviewer comments:

Reviewer #1

(Remarks to the Author)
Thompson and coworkers revised the manuscript extensively in response to the initial review, including additional experiments that help to address many of the concerns raised by the reviewers. The extent of the anatomical segregation of the afferent inputs from the two cortical regions is now clearer, and many of the comments were addressed by revisions to the manuscript text. The new figures are also clearer.

Major Comments

1. However, while the response to concerns about the behavioral experiments provides new and clear information about how the SNr target neurons contribute to activity in the open field, these findings do not address the specific role of the M1 and M2 efferents themselves. Specifically, the new DREADD approach for the open field experiments only involves inhibition of PV neurons that receive inputs from M1 or M2? There is no manipulation of the activity of the afferents themselves. Thus, this says nothing about the role of the two cortical inputs. The observation that the PV-expressing neurons targeted by M1 and M2 have different behavioral roles is useful, but these neurons may have other phenotypic or innervation differences that account for their differential responses to DREADD modulation. SNr PV neurons receive diverse inputs from multiple brain regions (Sabatini paper, https://www.nature.com/articles/s41593-020-00712-5) contributing to topographical and functional diversity. Thus, the observed behavioral effects could arise primarily from modulating lateral versus more medial SNr PV neurons, independent of the specific cortical (M1 vs. M2) inputs. It is unclear if M1→SNr(PV) and M2→SNr(PV) pathways have truly pathway-specific functional roles compared to global inhibition of PVs in those regions.

It would be useful to target the specific cortical inputs, and the best way to do this might be an optogenetic approach using the same dual AAV strategy to express arch or another inhibitor opsin in cortical neurons projecting to specific SNr PV+ neurons with optical fibers placed in the SNr. Even the original PdCO approach might work if the expression and fiber location were specific. These optical approaches would also allow for precise temporal control of afferent input.

2. Although the PV neurons predominate in SNr, they are mostly in the mid- and lateral-SNr. The authors need to acknowledge this limitation. The PV neurons are generally expressed within the regions shown to be innervated in Figure 5c, but Figure 5d indicates that M2 efferents also innervate the most ventromedial edges of the SNr where PV neurons are sparser.

3. The caption for Figure 6 indicates that panel 6b should include two images (left: M1 input, right: M2 input). The M2 image appears to be missing, which is likely an oversight on the part of the authors. In addition, the physiological data and Figures 5a and 5g indicate that M1 projections primarily target lateral SNr PV neurons, whereas M2 projections preferentially target medial SNr PV neurons. However, Figure 6b (which currently shows only a single image) and Figure 5e (where only a few labeled neurons are visible) are somewhat confusing and do not clearly support this distinction. Clarification and/or additional representative images would strengthen this point.

Minor comments:

• In the open-field behavioral experiments, the time interval between CNO injection and behavioral testing is an important experimental detail that needs to be specified.

• Providing the product number for CNO would be helpful, as both water-soluble and non–water-soluble formulations are commercially available. Similarly, several other drugs listed for physiology experiments in the Methods section lack product numbers and should be specified for reproducibility.

• In Extended Figure 7, the Keypoint-MoSeq analysis following M2-innervated PV neuron inhibition shows an increase in slow movement components and a decrease in head movements within specific behavioral syllables. Given the depth of this analysis, it would be useful to include a more explicit description of these behavioral changes, either through clearer textual descriptions or reference to representative video examples.

Reviewer #2

(Remarks to the Author)
The authors conducted additional experiments and extensively revised the manuscript. The authors have addressed all my concerns. I endorse the publication of this work. thanks.

Reviewer #3

(Remarks to the Author)
I co-reviewed this manuscript with one of the reviewers who provided the listed reports. This is part of the Nature Communications initiative to facilitate training in peer review and to provide appropriate recognition for Early Career Researchers who co-review manuscripts.

Reviewer #4

(Remarks to the Author)
The authors have convincingly addressed my concerns in this very fine work.

Reviewer #5

(Remarks to the Author)
The authors have made major improvements to the manuscript, addressing most of my concerns, while also excluding some previous experiments which had raised concerns. Of note, however, the exclusion of the optogenetic behavioral experiment also somewhat weakens the claims they can and do make.

Some concerns remain:

1. Behavior
1.1 They have replaced the previous behavioral experiments with new, more specific chemogenetic inhibition experiments and added additional behavioral analysis. At the same time, they have excluded the previous optogenetic terminal silencing experiments. While the new experiments more clearly show that the M1/M2-recipient SNr neurons can exert distinct behavioral effects, these experiments do not speak to the physiological role of the cortical inputs to these SNr neurons. The authors mostly phrase their results carefully to only talk about the role of these neuron populations, not their inputs (but see below). However, an expansion of the optogenetic terminal manipulation experiments would have allowed them to actually speak to the role of the inputs – without these, there is now no test of the behavioral role of these inputs, reducing the overall insights gained from their experiments.
1.2 While they are mostly careful in how they describe their results, there are a few instances which still imply that they show a behavioral role for the cortical inputs (instead of for the recipient neurons) and need to be changed:
1.2.1 Lines 84-85: they should be more precise here, stating that only the recipient neurons are chemogenetically inhibited, not the 'pathways'
1.2.2 Line 337: 'To probe the functional role of corticonigral transmission without interfering with collateral targets,…' - that is not what they are doing with these experiments – they do not probe the role of the corticonigral transmission, only of the recipient neurons.
1.2.3 To make things clear, they should add a clear disclaimer in the discussion that their experiments only speak to the role of the recipient neurons, not of the cortical inputs.
1.3 In their discussion, they suggest that the decreased locomotion with inhibition of M1-recipient SNr neurons may be due to a shift away from locomotion and towards fine motor control. However, their results do not seem to support this – they show more head movements (Ext. Fig. 7c), but actually reduced Head&Forepaw movements – which likely would include more fine motor control elements. Their behavioral readouts (in an open field) may not be suited to actually tease this apart, but they should make it clear in their discussion that they don't see indications for a shift towards fine motor control. In addition, they should provide videos of animals when they do not locomote – so that the reader can get an impression how this immobility looks like.
1.4 The behavioral effects of the inhibition of M1-recipient neurons show a clear trend, but are also somewhat variable, with some animals seemingly having the opposite effect (increased displacement and fraction of time mobile; Fig. 6e,f). Could this behavioral variability be due to variability in the injection sites? The authors should provide more information about their injection sites (see also my histology comments). Here, they could potentially show the average or overlaid injections for animals showing a decrease vs. those showing an increase (both for M1 and SNr injections).

2. Histology
2.1 I asked them in the first review to show the injection sites for at the very least all their tracing animals – which they did not do. While they state in their rebuttal that they used the same injection sites for all animals (obviously, but injections can be variable nevertheless), my point is that differences in injection sites in M1 and M2 may lead to different projection patterns in the SNr. Please show the injection sites for all tracing animals (all animals in Fig. 5) and the corresponding pattern in the SNr in the supplement, not just the examples in the main figures.
2.2 Fig. 5d: the density plot for the M2-efferents does not seem to resemble the image in Fig. 5c. Again, showing the individual animals may help to explain this discrepancy
2.3 Fig. 5h: they should show an overlay of the density plots to make it easier to see the differential distributions

Minor:
- Line 1060: '…was injected in the ipsilateral SNr…' – This gives the impression the injections for the behavior experiments were unilateral – this sentence should be clarified.
- Fig. 6b: They only provide the image for the M1 injections, but the legend says there should also be an image for the M2 injection. They should add that M2 image.
- Line 1071/1072 – 'left' and 'right' are mixed up here
- Line 1047: should be 'mCherry or GFP'

- Please add a note in the text that ‘n’ refers to cells and ‘N’ to animals
- Line 189: should be ‘characteristic’
- Lines 294-299: add the reference to the figure panels which show the mentioned results
- Line 311: GFP-positive and mCherry-positive?

Version 2:

Reviewer comments:

Reviewer #1

(Remarks to the Author)
The authors have addressed all remaining concerns in the study. In the final analysis it does appear that the suggested optogenetic strategy will not work given concerns about alteration of collateral afferents to SNc, even with careful optical probe placement. This reviewer has no additional concerns or comments.

Reviewer #3

(Remarks to the Author)
I co-reviewed this manuscript with one of the reviewers who provided the listed reports. This is part of the Nature Communications initiative to facilitate training in peer review and to provide appropriate recognition for Early Career Researchers who co-review manuscripts.

Reviewer #5

(Remarks to the Author)
The authors have addressed almost all of my concerns, and the manuscript has clearly improved. I think this is an important contribution and should be published.
I have one remaining comment (related to my point 1.4 from the previous round): I am surprised by the strong hesitancy of the authors to show their actual injection sites. While they have now added the injection sites and projection patterns for the tracing (Ex. Fig. 6), they are still not showing the injections for the behavioral animals in Fig. 6. While they don’t have the injections sites for all animals (some were used for validation of the silencing), they could have shown the available injection sites and not just the few examples in Ex. Fig. 9. While I don’t think this should keep the paper from being published, I am surprised – for a paper which makes a big point about topographic projection patterns, it seems important to actually show the injection sites, since what is actually being hit may strongly affect the outcomes of the experiments. Since they claim that all injection sites have been verified (although they themselves admit that this is not quite correct – they apparently did not verify the cortical injection sites for the animals which were used to verify their DREADD approach – see response to my comment 1.4 from last round), they should also be able to show them. I would strongly recommend for the future to show all injection sites, especially if topography is an important aspect of the studied circuits and outcomes (e.g., behavioral changes) are variable.

Open Access This Peer Review File is licensed under a Creative Commons Attribution 4.0 International License, which permits use, sharing, adaptation, distribution and reproduction in any medium or format, as long as you give appropriate credit to the original author(s) and the source, provide a link to the Creative Commons license, and indicate if changes were made.
In cases where reviewers are anonymous, credit should be given to 'Anonymous Referee' and the source.
The images or other third party material in this Peer Review File are included in the article's Creative Commons license, unless indicated otherwise in a credit line to the material. If material is not included in the article's Creative Commons license and your intended use is not permitted by statutory regulation or exceeds the permitted use, you will need to obtain permission directly from the copyright holder.
To view a copy of this license, visit https://creativecommons.org/licenses/by/4.0/

We thank the reviewers for the constructive comments and the overall support for our study. We have now revised the paper, with new experiments, further analysis, and textual changes. The changes introduced into the text are indicated in blue in one version of the main text. The new experiments include:

- New behavioral experiments using an intersectional Cre/Flp approach to study the role of cortically-targeted SNr neurons (new Figure 6 and Extended Data Fig S7)
- Anatomical characterization of topographically organized M1 and M2 projections onto distinct populations of SNr neurons (new Figure 5)
- Additional characterization of the synaptic input from M2 onto SNr neurons (new Figures 2-4). Figures 2 and 3 have switched places from the initial manuscript to the revised version.

All revisions are detailed below in a point-by-point reply to the reviewers' comments.

Reviewer #1 (Remarks to the Author):

The study by Thompson and coworkers provides strong evidence for monosynaptic glutamatergic inputs to the substantia nigra pars reticulata from projection neurons in both the primary and secondary motor cortices. The anatomical connectivity and role of these inputs and the targeted neurons in spontaneous locomotion were also examined. The authors used an impressive combination of anatomical, physiological, chemogenetic, optogenetic and behavioral experiments to provide this new evidence of the function of a cortico-nigral "ultradirect" pathway. They also took advantage of existing imaging databases to chart the collateral projections of these cortical neurons. Overall, the manuscript investigates a relatively understudied yet important neural circuit component within the basal ganglia using an ambitious framework. Previous studies had only provided anatomical evidence for this connection, and thus the convincing electrophysiological evidence for the monosynaptic cortico-nigral synapses is valuable to the field. Given that the SNr integrates projection inputs from multiple brain regions, dissociating the specificity of its functional role or downstream targets is a significant challenge.
The study provides valuable new information that will force a re-evaluation of key aspects of basal ganglia function. The roles of these projections in spontaneous locomotion is an important aspect of the study. If the results are supported as currently presented, this suggests interesting contributions to action initiation or termination (but see major comment 1 below). The discussion includes an informative consideration of how cortico-nigral connections could contribute to basal ganglia function and movement control. The possibility of convergence with other afferents innervating SNr is an interesting topic nicely discussed by the authors. While we acknowledge the importance of this scientific question and the authors' efforts, we find that the data

presented in the manuscript are insufficient to provide readers with a comprehensive understanding of the circuit in question. Some findings are overinterpreted given the limited supporting evidence. In particular there are concerns about the evidence for anatomical segregation of the M1 and M2 inputs to SNr, and the behavioral data lack sufficient quantification and completeness to clarify the specific role of these inputs in locomotion.

We thank the reviewer for the support and insightful comments, which we addressed in the revised manuscript, specifically addressing the comments regarding behavior and M1/M2 topography.

Major Comments

1. The caption for figure 6 appears to indicate that only 2-3 animals were examined in the chemo- and optogenetic experiments. Furthermore, the graphs in figure 6d,e,i and j do not indicate if there were significant differences across the groups (and the data only show mean and error bars with no points showing individual values in the different groups). Is this accurate? If these sample sizes are correct, then these experiments are woefully underpowered and the experiment needs to be repeated with reasonable samples sizes. Furthermore, it would be useful to show the individual values for each group so that the readers can appreciate the true range of values.

We fully agree with this comment and have now redone the behavioral part of the study using a more precise viral approach and higher numbers. In these new experiments we use Cre/Flp combinatorial viral injections to selectively manipulate corticonigral recipient inhibitory PV neurons in SNr. The new data is presented in the revised Figure 6, showing the impact of both M1/M2 recipient SNr neurons (specifically PV+ neurons of SNr) on behavior.

2. In addition, in figure 6d it appears that the GFP group shows greater distance traveled in the saline condition as compared to the other two groups. Could there be an effect of expression of the hM4D(Gi) construct itself, independent of agonist treatment?

In the new set of experiments (described above) we show the effect of CNO on the open field locomotion in mice expressing hM4D(Gi) and those not. There is a slight difference in the baseline conditions, however, chemogenetic inhibition showed significant (and opposing) behavioral effects in M1 and M2 transfections.

3. Could additional analyses help to clarify the role of the cortical-SNr projections in spontaneous locomotion. For example, do animals move for shorter periods of time or simply not initiate as many movement bouts when the neurons or projections are inhibited? Deeper analysis of this behavior might help to reveal what aspects of movement are likely to involve the cortico-nigral projections.

To address this comment, we have now extended our behavioral analyses. We quantify several additional behavioral properties in the freely moving mice, including an analysis of locomotor bouts and segmentation of behavioral “syllables” using Keypoint-Moseq (Weinreb et al., 2024). The analysis shows significant and opposing effects of the M1 and M2 pathways and is now presented in Figure 6 and Extended Data Figure 7 of the revised manuscript.

4. The topographical segregation of M1 and M2 projections in the SNr is a central claim of the manuscript. However, the authors only present limited data directly supporting this claim. For example, they do not show images or analysis of M2-SNr terminals from the experiments in which ChR2 was injected into M2, and the image in figure 6g appears to show only sparse M2 innervation around the SNr. Regarding the M1 projections, figure 1b appears to show relatively dense terminal labeling, but a lower magnification image is needed to show where this is within SNr. Likewise, the somatic labeling in figure 5b is not dense and does not appear to adequately represent the topographical features of the M1/M2 projections in the SNr as described in the text and figure 5d and e. The authors should show the images from all injected animals in the supplement for both the ChR2 and anterograde labeling experiments to provide more detailed information about innervation.

Following these comments we have performed a new set of injections to quantify and better show the respective topographic arrangement of M1/M2 projections in SNr. These injections include an intersectional viral approach using Cre/Flp injections in M1/M2 and dual color labelling of the respective recipient neurons in SNr. These new data are now presented in figure 5, supporting the topographic organization (we do not claim a complete “segregation” but different overall distribution) of M1 and M2 projections in SNr. Moreover, even in overlapping projection regions, the fraction of neurons receiving both M1 and M2 input is tiny. In the new Figure 5 we show examples and a summary of all data (Figure 5e-h).

5. More information is also needed about the viral scheme and graphical approach used in Figures 5d and e. The manuscript must clearly detail the methods for data collection and processing. Furthermore, there is a significant discrepancy between the raw fluorescence image in Figure 5c and the processed data in Figure 5e unbiased

data analysis and statistical validation of Figure 5e are necessary to support the claim of spatial segregation.

Our findings do not support a complete separation in the projection fields from M1 and M2 to the SNr. In the text we are careful to state ‘topographical organisation’ and not ‘segregation’ as there is a degree of overlap in the projection fields. Figure 5 has now been updated to show dual Cre & Flp injections in M1 and M2, with a single injection in the SNr containing both Cre- & Flp-dependent fluorophores, showing that despite the overlap in axonal projection fields there is a high degree of separation between the two recipient SNr populations. Cell counting was performed blindly and statistical validation of topography performed by Hotelling’s T2 statistical test.

6. A straightforward and effective way to demonstrate the segregation of projections would be through a two-color viral injection (e.g., GFP in M1 or mCherry in M2) to visualize the fluorescence distribution of afferents and terminals in the SNr. It would be beneficial to use FLP and CRE based schemes to image both SNr/M1 and SNr/M2 in the same brain, if possible. As it stands, the manuscript does not provide essential evidence to substantiate this key assertion.

We thank the reviewer for these comments. In the revised version we have now followed these suggestions, using both dual-color anterograde labeling from M1 and M2, as well as a Cre/Flp approach for transsynaptic labeling (Figure 5).

7. Dopaminergic neurons could be directly activated by motor cortex projections (Whole-Brain Mapping of Direct Inputs to Midbrain Dopamine Neurons, M. Watabe-Uchida et.al , Neuron, 2012), although admittedly few such connections were observed in this study. However, these sparse connections could still contribute to the findings in Figures 6f-j. The fluorescence vacancy observed in Figure 6g, particularly in the SNr, raises further
concerns. While the optogenetic manipulation of M1/M2 terminals is an interesting approach, this manipulation could potentially lead to activation of both SNr neurons and dopaminergic neurons. I believe expressing an inhibitory opsin in the SNr or specifically in SNr-projecting cortical neurons would be a valuable additional approach.

This is an important point, which was a strong motivation for a whole new set of experiments. We addressed this issue in the revised paper by using the intersectional Cre/Flp approach, allowing for selectivity in the targeting of PV+ postsynaptic cells in SNr. We have now redone the behavioral part of the study using the intersectional viral approach and higher numbers. In these new experiments we use Cre/Flp combinatorial viral injections to chemogenetically manipulate corticonigral recipient inhibitory PV neurons in SNr. The new data is presented in the revised Figure 6, showing the impact of both M1/M2 recipient SNr neurons (specifically PV+ neurons of

SNr) on behavior. Regarding the "fluorescence vacancy": the high density of labelled fibers passing around the SNr via the internal capsule and cerebral peduncle causes the SNr to appear comparatively dark. We have further characterized the innervation of the SNr by M1/M2 terminals and included clear examples of cortical-efferent terminals in Figure 5.

Minor Comments

i. Can the authors provide any information about the cortical layers that contain the somata of the cortico-nigral neurons? One would presume that these are deep layer neurons, but do they arise from layer V or VI?

We did not label the corticonigral populations retrogradely, however, the population of SNr-projecting cortical neurons has been labelled in Foster et al., (Nature, 2021) and Munoz Castenada et al., (Nature, 2021), showing PT pyramidal cells primarily in layer 5 (upper and lower). We now mention and discuss this in the revised manuscript (page 12 line 437).

ii. The examination of potential S1 cortex-SNr projections is a useful part of the story. It would be helpful to know if other cortical regions project directly to SNr, but admittedly this is beyond the scope of the present study. This possibility should be discussed however, and it would be helpful to know if the literature suggests that any other cortical neurons project directly to SNr. This would help readers to evaluate if this is a unique property of the motor cortices.

Mapping the entire cortical sheet for SNr projections is indeed beyond the scope of this study. However, we discuss the possibility and existing evidence for different and parallel corticonigral projections (See discussion).

"...In this study we focused on only two cortical regions of the corticonigral pathway (M1 and M2) and showed the lack of such innervations from S1 (extended figure S3). It is, however, possible that other cortical regions also project to SNr, as was recently shown for insular cortex[57]..." (lines 338-392)

iii. Figure 6b, (DIO-hM4Di expression), if the authors applied the same cell analysis technique used in Figures 5d-h (DIO-eGPF) , would they reach the same conclusion?

In the new Cre/Flp experiments (revised Figure 5) we used an anterograde transsynaptic dual color approach that provides simultaneous labeling of M1 and M2 recipient SNr cells. The overall distribution of postsynaptic cells agrees with the observed axonal projection field in SNr (Figure 5C). Similar results are observed qualitatively with expression of hM4Di-mCherry, however we did not apply the same quantitative analysis.

iv. From the histology analysis, what percentage of SNr neurons are directly innervated by either M1 or M2? If this percentage is low how can they exert a dominant influence on locomotion while other functionally active neurons in the SNr are present?

The percentage of cortical recipient SNr cells is not so straightforward to quantify just from the histological data. Rather, the synaptic responses recorded using patch-clamp electrophysiology may provide a better assessment, showing clearly that most SNr neurons within the respective projection field receive M1 or M2 cortical inputs (See Figures 1-3). Furthermore, the SNr exists as a severe bottleneck in the cortico-basal ganglia circuit. The output of millions of neurons is effectively funneled down to less than twenty thousand cells in the SNr. These circuits also appear to be extremely parallelized (see e.g., Foster et al., 2021), suggesting limited redundancy in their roles. We expect that manipulating distinct populations of SNr neurons can introduce biases in behavioural output. Our behavioural data does not support a ‘dominant influence', but rather a bias towards/against locomotion.

v. Figure 6f, if the behavioral indicators are collected over the entire 300-second period (x4), is it possible to provide a more detailed time course of locomotion before and during the optogenetic light exposure, rather than just 3 time points?

In the new set of behavioral experiments we used the chemogenetic approach only, where we express inhibitory DREADDs in PV-positive SNr neurons receiving monosynaptic connections from M1 or M2. This approach allowed us to avoid off-target effects of optogenetic terminal stimulation on other cell populations in the midbrain. We therefore decided to exclude the optogenetic experiments due to their lack of specificity and focus on the updated chemogenetic approach with higher numbers and selective targeting of SNr neurons.

vi. Figure 4a, the action potentials during the 20Hz stimulation are very tightly packed and some appear to have decreased amplitudes. Perhaps the authors should include an inset with an expanded timescale time axis so that individual spikes can be more readily distinguished.

The varying spike amplitudes in that figure were due to low sampling rate, however, they have no impact on the calculated spiking frequency (i.e., no spikes have been missed or added). We repeated these experiments with higher sampling rate, and replaced the example traces in the figure for clarity.

vii. Page 14, the authors should indicate the GFP vector that was injected as the control for the chemogenetic experiments.
This was originally indicated in text "*For control experiments, AAV2-CAG-GFP (Addgene #37825; Penn Vector Core, U.S.A.) was injected in place of the opsin*." However, this has now been updated to reflect the vector used in the new experiments (lines 544-546).

viii. Page 9, 1st line, the authors refer to the parvicellular reticular nucleus. Should this be parvocellular? Two lines later, perhaps "pontine" should be "pons"?

Corrected in the revised manuscript.

Reviewer #2 (Remarks to the Author):
The work by Thompson et al demonstrated a monosynaptic pathway from the motor cortex to the substantia nigra pars reticulata (SNr), which is the main basal ganglia output nuclei in rodents. This work is timely and important to the basal ganglia field because the direct cortico-nigral pathway provides cortical access to the output structure of the basal ganglia by passing the traditional basal ganglia direct and indirect pathway models. The work is original, rigorously designed, and well conducted and will advance our understanding of cortico-basal ganglia circuits in regulating motor and nonmotor function. I only have a few minor comments that may help to further consolidate the conclusion of this study.

We thank the reviewer for the positive feedback and comments.

1. The existence of cortico-nigral projection can be further strengthened by additional histology studies. For example, quantification of the presence of the vesicular glutamate transporter 1 (vGluT1) immunoreactivity in the SNr will complement with the existing electrophysiology data. It would be even informative to compare the relative density of vGlu1 puncta versus vGluT2 puncta in the SNr, providing an estimated relative innervation of SNr neurons by the cortical inputs versus subthalamic inputs.

The existence, distribution and quantification of VGluT1 and VGluT2 puncta in the SNr has been assessed previously (Kessler et al, Glia 2020, Martin-Ibanez, EJN 2006). However, they were not directly attributed to cortical projections. While we have not counted the VGluT1/2 distribution directly, we show that the corticonigral pathway is robust, using direct synaptic measurements from both M1 and M2, as well as the morphological and transsynaptic labeling. Moreover, the SNr receives glutamatergic input from multiple sources, including the medulla, PPN, and more, all of which are likely sources of VGluT2 expression in addition to STN projections.

2. Does the globus pallidus internal part (GPi, another basal ganglia output nuclei in rodents) also receive monosynaptic inputs?
3. The existence of direct projection from the cortex to the basal ganglia output nuclei was indicated in the literature. The work by Milardi et al (2015) (PMID: 25156805) should be discussed.

These are interesting points, especially given the recently reported reciprocal connectivity between the cortex and GPe [Ferenczi et al., (2025)], and our current study. We have not tested the cortico-GPi pathway here, as it would require a complete duplication of our study and is beyond the scope of this study. However, cortical connections to GPi have indeed been described previously in rodents by Naito & Kita 1994. As mentioned by Milardi et al., 2015, there are also homologous projections in lamprey, monkeys and humans. These are now discussed in the revised version (page 12) in the context of cortico-pallidal connections.

"Cortical projections also target the globus pallidus externa and interna, both of which complement the SNr as elements of the basal ganglia output stage [30,72–75]. GPe-projecting cortical neurons show diverse projection patterns, with some neurons collateralizing in the SNr. One electrophysiological study in rats found that, within the GPe, input from motor cortices was biased towards the striatum-projecting arkypallidal neurons, which do not project to the SNr30. In contrast, multiple studies in mice have reported cortical input to the SNr-projecting prototypic neurons of the GPe, as well as the arkypallidal cells[29,57,74]. Corticopallidal projections may serve a similar function as corticonigral projections: bypassing the striatum for direct access to basal ganglia outputs." (lines 426-435)

4. The data in Figure 2 showing the lack of NMDARs-mediated glutamatergic transmission are surprising and unexpected. What does it mean in terms of synaptic plasticity and motor learning or general motor control? In the absence of postsynaptic NMDRs, do the cortico-nigral synapses functionally express Ca2+ permeable AMPARs? The subthalamus nucleus (STN) provides the major excitation to the SNr GABAergic neurons. Does the STN-SNr transmission involve NMDARs? Further experimental validation and clarification on NMDAR-mediated EPSC/EPSP may help to address potential technical issues during the recording.

We thank the reviewer for this comment and have therefore added more experiments showing **1)** the lack of a significant NMDA component in the corticonigral synapses, and **2)** the existence of a Ca^{2+} permeable AMPA receptor in this pathway (See revised Figures 3 and extended data figure 4). The lack of NMDA component is, in fact, not so unusual and has been seen also in the corticostriatal projections onto PV-interneurons both in the dorsal striatum (Johansson & Silberberg 2020), and in the somatosensory cortex (Morabito et al., 2025). Subthalamic inputs to the SNr (Sitzia et

al, 2022) are predominantly mediated by AMPA receptors, with a small NMDA component. While the characterization of STN-SNr synapses is beyond the scope of this paper (and well described already), it has been previously shown that the small proportion of NMDA receptors that do exist at the STN-SNr synapse can mediate synaptic plasticity in this pathway (Dupuis et al, (2013)).

5. It would be helpful to clarify how much overlap between cortico-nigral pathway and corticospinal/cortical-STN projections in rodents. See Milardi et al (2015) (PMID: 25156805) for additional information in human.

Indeed, the cortical cells projecting to SNr have other targets and axon collaterals, as seen in Figure S6 and previous reports (Economo, et al., 2018; *BICCN*, 2021)These include cortical projections to the striatum, brainstem, and spinal cord, among other targets. The paper by Jeon et al., 2022 shows sparse SNr targeting compared to massive STN projections (the complementary experiment to ours). The Milardi paper is now discussed in the context of cortical inputs to other basal ganglia nuclei, as indicated above.

Reviewer #3 (Remarks to the Author):

I co-reviewed this manuscript with one of the reviewers who provided the listed reports. This is part of the Nature Communications initiative to facilitate training in peer review and to provide appropriate recognition for Early Career Researchers who co-review manuscripts.

Reviewer #4 (Remarks to the Author):

Thompson and colleagues conducted a compelling series of experiments demonstrating the cortico-nigral pathway. They used anatomical, electrophysiological, chemogenetic, and optogenetic techniques to elucidate this pathway both structurally and functionally. Their approach was thorough and their findings were convincing. This work adds a new and important pathway to the basal ganglia circuit, one likely to have behavioral and clinical relevance.

The majority of their experiments were fastidiously executed, addressing any concerns I might have had as I was reading through the Results. The one exception was the final pair of experiments, the behavioral studies.

1. The behavioral studies. In the abstract, they're a good pair of experiments: you modulate the presynaptic side of the pathway, you modulate the postsynaptic side of the pathway, and the results end up being consistent with each other. But the problem I see is that the SNc could have been affected in both experiments, and that could explain the results. Dopaminergic neurons of the SNc receive cortical input from M1 and M2 (Watabe-Uchida et al 2012 Neuron), dopaminergic neuron activity increases during movement (Schultz et al 1983 Exp Brain Res), and inhibition of SNc neurons is aversive (Ilango et al. 2014 J Neurosci), which seems like it could lead to akinesia during inhibition. And since the SNr and SNc are right next to each other, it's hard to affect one without affecting the other. Unless more is done to address this alternative, the cortico-SNr pathway can't be described as the sole mediator of the behavioral effects you recorded.

This is an important point. We have therefore performed a new set of behavioral experiments using a selective conditional Cre/Flp viral approach. These experiments enable us to manipulate only the PV-expressing SNr cells receiving the cortical inputs. We have also increased the number of animals in these experiments, now presented in Figure 6 in the revised manuscript.

1a. In the DREADD experiment, did any dopaminergic neurons of the SNc express the receptor? When I looked carefully at Fig. 6b, I didn't see any, but were all sections free of receptor expression in the SNc? Were all receptor-expressing neurons TH negative?

This point is addressed by the new behavioral experiments (Figure 6). In the new set of behavioral experiments, we used a chemogenetic approach, where we express inhibitory DREADDs in PV-positive SNr neurons receiving monosynaptic connections from M1 or M2. This approach allowed us to avoid off-target effects of optogenetic terminal stimulation on other cell populations in the midbrain. We therefore decided to exclude the optogenetic experiments due to their lack of specificity and redo the chemogenetic experiments with higher numbers and selective targeting of SNr neurons.

1b. The optogenetic experiment may also be subject to the same issue of potentially affecting the SNc. In Fig 6g, the tip of the optic fiber appears to end right in the middle of a big bundle of opsin-expressing cortical axons that pass through the SNc. Can you offer evidence that only or mostly the SNr was affected in this experiment? In your trans-synaptic labeling experiments (from Fig. 5) did you see any labeling of SNc neurons?

This is an important point related also to the points above. Indeed, we cannot rule out potential effects of direct cortico-SNc manipulation. We therefore performed a new

set of experiments using the Cre/Flp approach detailed above, which ensured selective targeting of cortically-recipient PV+ cells in SNr.

1c. Also, were the DREADDs and opsins expressed unilaterally or bilaterally? I saw in Methods your given surgical coordinates were unilateral, so I assume your interventions were unilateral, but clearly stating that in the body of the paper would be helpful. Unilateral manipulations of the basal ganglia in movement studies have a tendency to make the animal rotate or walk in circles, rather than exhibit strict immobility (e.g., Chen, Chan & Yung 2002 Neuroscience), so your behavioral findings, if unilaterally generated, would stand out.

All new experiments (described above and in the new Figure 6) are now done using bilateral injections.

Minor points/questions.

1. What wattage of light was used in the electrophysiological recording experiments?

This is indicated in the figure legends but has now been added to the methods section as well (1 mW).

2. The 'widefield photostimulation' of M1 and M2 was aimed where: the whole slice, M1/M2, or SNr?

Photostimulation in the *ex vivo* experiments was done above the recorded cells in SNr, to activate the corticonigral synaptic terminals. This has now been clearly indicated in the methods.

3. In Figure 2d the labels on the pie chart are unclearly placed.

Fixed in revised manuscript

4. In Fig. 5d the brain is a little hard to see.

Fixed in revised manuscript

5. In the section, "Photoactivation of motor cortex axon terminals increases SNr firing rate ex vivo," were coronal or sagittal slices used (or both)? The Methods for the recording experiments states that slices were either coronal or sagittal. In sagittal slices it is possible that the whole cortico-striatal-nigral pathway is intact, and stimulation of channelrhodopsin-expressing terminals in the nigra could antidromically activate cortical axons throughout the whole descending pathway, indirectly

stimulating other components of the basal ganglia like the STN. Also clarify whether drugs were added to the solutions to record monosynaptic activity only. Again, I know the Methods describes that such drugs were used at times, but adding it to the Results section makes it unequivocal, and I don't have to go digging around in the Methods section for that detail.

We thank the reviewer for these comments. The pharmacological manipulations are now better described also in the results section. Specifically, gabazine was bath-applied in order to block any polysynaptic GABAergic responses to photostimulation. The recordings presented in Figures 1-4 were done in both parasagittal and coronal slices, which is now explicitly reported (Extended Data Figure 5). It is not very likely that the entire polysynaptic pathway is maintained even in parasagittal slices, but we have now included a comparison between cell-attached recordings from coronal and parasagittal slices showing no difference in SNr firing rate increases with cortical terminal stimulation (Extended Data Figure 5).

Reviewer #5 (Remarks to the Author):

This manuscript by Thompson et al. sets out to test whether the previously described, but hardly explored projections from motor cortex (primary M1 and secondary M2) to the substantia nigra pars reticulata (SNr) are functional, whether projections from M1 and M2 target distinct SNr neuron populations and whether their manipulation has behavioral effects. For this, they optogenetically activate axon terminals of M1 or M2 neurons in SNr while patching SNr neurons. They found that about half of the neurons receive monosynaptic AMPA-mediated inputs. They further show that M1- and M2-targeted SNr neurons have some distinct properties (firing threshold, spontaneous firing rate). Furthermore, they show that the motor cortex to SNr synapses have mixed properties, some showing facilitation, some depression in the paired pulse ratio. They also show in cell-attached ex vivo recordings that motor cortex inputs can modulate the firing of targeted SNr neurons. Anatomical tracing using anterograde AAV1s shows that M1- or M2-targeted SNr neurons have distinct projection patterns to other subcortical areas. Taken together with their patch results, this suggests that M1 and M2 target distinct populations of SNr neurons. Finally, the authors show experiments suggesting that the M1/M2 to SNr projections can affect spontaneous behavior – silencing of M1 or M2 axon terminals in SNr or of targeted SNr neurons, using either opto- or chemogenetics, reduces the distance travelled in an open field test.
The characterization of the previously described, but largely unexplored projections from motor cortex directly to the SNr is important and of high interest, since these projections add another control pathway via which cortex may directly influence motor behavior – either in coordination with or bypassing the upstream nuclei of the basal ganglia. The combination of careful in vitro characterization of the connectivity, anatomical tracing and behavior is powerful. Especially the in vitro patch clamp experiments are rigorously done. However, an incomplete description/

characterization of their injection sites and tracing as well as very preliminary behavioral results dampen the enthusiasm. Below are my individual concerns:

Major:

- Behavior: The animal numbers for the behavior are too low to draw any reliable conclusions. They have groups of only 2-3 animals for both their chemo- and optogenetic experiments. While the results show the trends they are describing, they are highly variable (even at baseline) and no statistics have been performed (also not meaningfully possible with such low n). Currently, the behavior results are not convincing, and additional animals need to be added.

This is an important point, raised also by other reviewers. We have now performed a new set of behavioral experiments using a selective conditional Cre-on Flp-on viral approach. These experiments enabled us to manipulate only the PV-expressing SN cells receiving the cortical inputs. We have also increased the number of animals in these experiments, now presented in Figure 6 in the revised manuscript.

- Behavior: They seem to only inhibit chemo- or optogenetically unilaterally. While bilateral inhibition would have likely provided clearer results, they should at the very least test whether these unilateral manipulations lead to any lateralized effects on behavior, e.g., turning.

The new experiments described above are all bilateral manipulations. Regarding more detailed behavioral assessment, please see the point below.

- Behavior: The analysis is rather superficial. While I understand that they try to just show that behavior is affected at all, it would be good if they could make more use of their data. In addition to looking at, e.g., turning (see above), they could also look at measurements like running bout number and length (indication for effects on action initiation), changes in center vs. wall locations etc.

We have now analyzed additional behavioral properties in the freely moving mice, including the frequency and duration of movement bouts, all of which are presented in the new Figure 6 and Extended Data Figure 7 of the revised manuscript.

- Behavior: they did not validate the PdCO approach – since axon terminal inhibition comes with caveats, it would have been good to see, at least in a slice, that their approach indeed does what it is supposed to do. I am aware that this tool has been published, but it has not been widely used, and its effects may vary across different types of projections

This is a good point. The pdCO approach is quite novel and indeed the results were based on a small number of experiments. Moreover, we could not rule out the transfection of non-GABAergic neurons, in either SNr or SNc. We therefore decided to focus on the new chemogenetic experiments using the selective Cre-on/Flp-on approach as described in the revised manuscript (Figure 6).

- Histology: They need to show/quantify their injection sites for all experiments. They make claims about specific targeting in SNr etc., but the observed patterns might be due to their specific injection sites. Controlling for this is especially important given that they claim a clear topography of M1/M2 inputs in SNr.

The viral injections were done into the coordinates specified in the methods (1.5 AP, -1.75 ML, -0.8 DV) for M1 and (2.3 AP, -1.0 ML, -0.9 DV) for M2). Injection sites are presented in figures 1,2, and 5. In the experiments shown in the revised manuscript (Figure 5) we use dual color labeling of M1 and M2 recipient cells (through a single SNr-targeted injection, reducing variation in injection site), which agree with the organization of the corticonigral projection fields.

- Histology: They do not specify their inclusion/exclusion criteria for any of their experiments – were animals with too big/ too small injections excluded? Did they quantify injection sites to determine exclusion?

Exclusion criteria for injections have now been made explicit in the methods section, on page 18 line 665.

- Tracing: Fig. 5 – their axon tracing for SNr neurons that are targeted by M1 or M2 seems preliminary. There are no quantifications, no comparisons across animals, etc. While the shown examples are intriguing, this seems insufficient. Especially combined with the lack of a presentation and quantification of the injection sites. Are the differences in M1 and M2 experiments maybe due to incomplete/variable injections?

- Tracing: Fig. 5 - they trace the axons of SNr neurons that are labeled using AAV1-Cre and AAV2-FLEX-GFP. It is important to confirm that there are no neurons labeled outside SNr – otherwise, the axons they trace in other areas might originate from such non-SNr neurons. From the one example image (Fig. 5c) it is hard to see, but already there may potentially be labeled neurons outside SNr.

- Tracing Fig. 5: related to above – does the AAV2-FLEX injection cover the same area in SNr across M1/M2 animals – otherwise, any difference may be misleading

We thank the reviewer for these comments. The injections in SNr were done in the same coordinates in all the tracing experiments. Indeed, due to the shape and position of the nucleus it is virtually impossible to completely cover the SNr without labelling non-SNr neurons. We could not rule out injection variability, while maintaining specificity. Therefore, we have omitted these experiments, and we focus on the topography of the corticonigral projection itself and the somatic distribution of cortical-recipient neurons in SNr (Fig. 5).

Minor:

- ED Fig. 1: they state in the legend they have recorded from 38 neurons in 51 animals. They should state the actual number of animals from which these neurons are recorded.

We have now added the exact numbers of respective recordings from M1 and M2 injected mice in the figure legends.

- Fig. 6a: Why does the scheme mention ‘ex vivo electrophysiology’?

We thank the reviewer for spotting this error; it has been removed in the revised version.

We hope that you will find the revised manuscript now in good shape and ready for publication.

REVIEWER COMMENTS

We thank the reviewers for the continued support for our study. We have made changes to the text to address the detailed comments below and have included new extended data figures, and videos, to support the main results, while addressing any concerns. The changes introduced to the text are indicated in blue in one version of the main text. All revisions are detailed below in a point-by-point reply to the reviewers' comments.

Reviewer #1 (Remarks to the Author):

Thompson and coworkers revised the manuscript extensively in response to the initial review, including additional experiments that help to address many of the concerns raised by the reviewers. The extent of the anatomical segregation of the afferent inputs from the two cortical regions is now clearer, and many of the comments were addressed by revisions to the manuscript text. The new figures are also clearer.

Major Comments

1. However, while the response to concerns about the behavioral experiments provides new and clear information about how the SNr target neurons contribute to activity in the open field, these findings do not address the specific role of the M1 and M2 efferents themselves. Specifically, the new DREADD approach for the open field experiments only involves inhibition of PV neurons that receive inputs from M1 or M2? There is no manipulation of the activity of the afferents themselves. Thus, this says nothing about the role of the two cortical inputs. The observation that the PV-expressing neurons targeted by M1 and M2 have different behavioral roles is useful, but these neurons may have other phenotypic or innervation differences that account for their differential responses to DREADD modulation. SNr PV neurons receive diverse inputs from multiple brain regions (Sabatini paper, https://www.nature.com/articles/s41593-020-00712-5) contributing to topographical and functional diversity. Thus, the observed behavioral effects could arise primarily from modulating lateral versus more medial SNr PV neurons, independent of the specific cortical (M1 vs. M2) inputs. It is unclear if M1→SNr(PV) and M2→SNr(PV) pathways have truly pathway-specific functional roles compared to global inhibition of PVs in those regions.

It would be useful to target the specific cortical inputs, and the best way to do this might be an optogenetic approach using the same dual AAV strategy to express arch or another inhibitor opsin in cortical neurons projecting to specific SNr PV+ neurons with optical fibers placed in the SNr. Even the original PdCO approach might work if the expression and fiber

location were specific. These optical approaches would also allow for precise temporal control of afferent input.

Both reviewers 1 and 4 noted in the previous review that one could not exclude a coactivation of cortical projections (M1 and M2) to dopamine neurons in SNc. As a direct response to this critique, we focused our revisions on specificity, using chemogenetic expression in specific PV+ target neurons of M1 and M2 in SNr, and observing distinct and opposite behavioral effects of inhibition of the two target cell populations.

While we attempted direct optogenetic manipulation of cortical efferents in the original submission, both reviewers 1 & 4 rightfully questioned the specificity of postsynaptic targets in the initial revision. Dopaminergic neurons, both within the SNr and in the overlying SNc, could be manipulated by any such experiment (see new supplementary Extended Data Fig. 7). Even with perfect fiber placement, dopamine-releasing dendrites of SNc dopaminergic neurons extend into the SNr, making them impossible to spatially disentangle *in vivo*. While a system to express an opsin specifically in SNr-targeting axons would be ideal, this is not possible with the AAV strategy mentioned. *Therefore, we conclude that the approach suggested in the review would not solve the problem of target specificity*. Based on these comments, we reasoned that the most specific experimental manipulation possible was to directly manipulate the post-synaptic PV+ SNr neurons. While this, as the reviewer has pointed out, is not a manipulation of the corticonigral pathway per se, it does show the action of the output neurons of the SNr that are the target of the corticonigral projections and provide an indication to the role of the pathway-defined subpopulations. We have changed the wording at several points in the text to clarify this (please see responses to reviewer #5 for specifics).

"Thus, the observed behavioral effects could arise primarily from modulating lateral versus more medial SNr PV neurons, independent of the specific cortical (M1 vs. M2) inputs. It is unclear if M1→SNr(PV) and M2→SNr(PV) pathways have truly pathway-specific functional roles compared to global inhibition of PVs in those regions."

PV neurons represent 86.9% of SNr neurons and the non-PV GABAergic neurons have the same projection patterns as the PV+ SNr neurons (McElvain et al., 2021). We also show (Fig. 5) that the M1 and M2 corticonigral projections target separate subpopulations of SNr cells. The overall effects of inhibiting either of these two subpopulations differ as shown by the chemogenetic experiments. However, within these two subpopulations of SNr neurons there will be a further subdivision of SNr neurons targeting different midbrain – brainstem motor centers (McElvain et al., 2021).

We agree that our behavioral results cannot establish distinct roles for the two types of cortical-recipient SNr neurons as compared to non-recipient neurons. Unequivocally establishing a neuron as non-recipient through viral strategies is significantly more challenging than accessing the recipient cells. Despite their utility, transsynaptic viral approaches still have

an inherent incomplete efficiency (Zingg et al., 2020). Our behavioral experiments focus on the roles of M1-recipient vs M2-recipient populations. We have been careful to clarify this in the text.

In addition, we fully agree that any functional differences may also be correlated with the position of the neurons along the ventromedial-dorsolateral axis (and accompanying gradients in afferent inputs). *We do not see this as a confounding factor, but rather a feature of the topography.* The topographical innervation of cortical afferents in the SNr described by both us, and others (Foster et al., 2021; Yang et al., 2023), *inseparably links the afferent input to the spatial location of a cell.* As the reviewer has pointed out, similar topography is seen in striatonigral projections (Deniau et al., 1996; Lee et al., 2020; Foster et al., 2021; Sitzia et al., 2024), with different input regions having different behavioral roles (Lee et al., 2020).

2. Although the PV neurons predominate in SNr, they are mostly in the mid- and lateral-SNr. The authors need to acknowledge this limitation. The PV neurons are generally expressed within the regions shown to be innervated in Figure 5c, but Figure 5d indicates that M2 efferents also innervate the most ventromedial edges of the SNr where PV neurons are sparser.

Most GABAergic neurons in the SNr are PV positive, including the vast majority of projection neurons (86.9% reported in McElvain et al., 2021). For that reason, we chose to focus on the PV+ population in our behavioral experiments. The density plots presented in figure 5d represented the cumulative axonal innervation across the rostrocaudal axis, including regions with varying degrees of PV expression. Moreover, our anterograde tracing does not differentiate between axon fibers and terminals. Axons passing through the cerebral peduncle, which may or may not collateralise in the SNr, are labelled by this method. These are especially prominent at the anterior-most end of the SNr. We have now updated figure 5 to show subdivisions along the rostrocaudal axis of the SNr. It is of course plausible that cortical projections also target non-PV neurons in SNr, including sparse innervation of SNr dopaminergic cells (Extended Data Fig. 1, 2). To this end, motivated by the initial reviewer comments, we have opted to prioritise target *specificity* over target completeness when performing behavioral manipulations.

This is now acknowledged in the discussion. as follows:

"In the behavioral experiments presented here (Fig. 6 and Extended Data Fig. 9, 10), we chose to manipulate PV-expressing neurons receiving input from either M1 or M2. The vast majority of GABAergic neurons in the SNr express PV, while the rest of the GABAergic SNr neurons share similar projection patterns (McElvain et al., 2021). Performing in vivo manipulations in an intersectional manner with PV-Cre mice was motivated so as to avoid the potential manipulation of other neuron types_in the vicinity of the SNr, most notably the dopaminergic populations of either the SNr or SNc."

3. The caption for Figure 6 indicates that panel 6b should include two images (left: M1 input, right: M2 input). The M2 image appears to be missing, which is likely an oversight on the part of the authors. In addition, the physiological data and Figures 5a and 5g indicate that M1 projections primarily target lateral SNr PV neurons, whereas M2 projections preferentially target medial SNr PV neurons. However, Figure 6b (which currently shows only a single image) and Figure 5e (where only a few labeled neurons are visible) are somewhat confusing and do not clearly support this distinction. Clarification and/or additional representative images would strengthen this point.

We have modified both figures according to these comments, showing clearer examples of the topography of postsynaptic SNr neurons. Please note that both Fig. 5f and Fig. 6b are examples of single sections and do not reflect the whole distribution of postsynaptic SNr neurons. We have changed the representative section shown in Fig. 5f and have added arrowheads to direct the reader.

Minor comments:

• In the open-field behavioral experiments, the time interval between CNO injection and behavioral testing is an important experimental detail that needs to be specified.

This time interval (30 minutes) is now indicated in the Methods section.

• Providing the product number for CNO would be helpful, as both water-soluble and non–water-soluble formulations are commercially available. Similarly, several other drugs listed for physiology experiments in the Methods section lack product numbers and should be specified for reproducibility.

Product numbers for all pharmacologic agents are now indicated in the Methods section.

• In Extended Figure 7, the Keypoint-MoSeq analysis following M2-innervated PV neuron inhibition shows an increase in slow movement components and a decrease in head movements within specific behavioral syllables. Given the depth of this analysis, it would be useful to include a more explicit description of these behavioral changes, either through clearer textual descriptions or reference to representative video examples.

We have now addressed this comment by adding video examples of mice in an open field

assay, demonstrating different behaviors during stationary periods (requested also by reviewer #5). We have also expanded the text in both the Results and Methods, further describing the observed behaviors. Please note that the previous Extended Data Figure 7 is now Extended Data Figure 10.

Reviewer #2 (Remarks to the Author):

The authors conducted additional experiments and extensively revised the manuscript. The authors have addressed all my concerns. I endorse the publication of this work. thanks.

Reviewer #3 (Remarks to the Author):

I co-reviewed this manuscript with one of the reviewers who provided the listed reports. This is part of the Nature Communications initiative to facilitate training in peer review and to provide appropriate recognition for Early Career Researchers who co-review manuscripts.

Reviewer #4 (Remarks to the Author):

The authors have convincingly addressed my concerns in this very fine work.

Reviewer #5 (Remarks to the Author):

The authors have made major improvements to the manuscript, addressing most of my concerns, while also excluding some previous experiments which had raised concerns. Of note, however, the exclusion of the optogenetic behavioral experiment also somewhat weakens the claims they can and do make.

Some concerns remain:

1. Behavior

1.1 They have replaced the previous behavioral experiments with new, more specific chemogenetic inhibition experiments and added additional behavioral analysis. At the same time, they have excluded the previous optogenetic terminal silencing experiments. While the new experiments more clearly show that the M1/M2-recipient SNr neurons can exert distinct behavioral effects, these experiments do not speak to the physiological role of the cortical inputs to these SNr neurons. The authors mostly phrase their results carefully to only talk about the role of these neuron populations, not their inputs (but see below). However, an expansion of the optogenetic terminal manipulation experiments would have allowed them to actually speak to the role of the inputs – without these, there is now no test of the behavioral role of these inputs, reducing the overall insights gained from their experiments.

Both reviewers 1 and 4 noted in the previous review that one could not exclude a coactivation of cortical projections to dopamine neurons in SNc. As a direct response to this critique, we focused our revisions on manipulating the specific target neurons of M1 and M2 in the SNr and recorded distinct and opposite behavioral effects of chemogenetic inhibition of these two target cell populations.

Since our previous optogenetic experiments suffer from the uncertainty of possible interference with the dopamine neurons in SNr or in the adjacent SNc, we decided to focus on the effects of the specific chemogenetic experiments. While expression of an opsin specifically in SNr-targeting axons would be ideal, this is not possible with the AAV strategy mentioned. *Therefore, we conclude that the approach suggested in the review would not solve the problem of target specificity*. However, the chemogenetic manipulation indeed has the inherent limitation that we do not test the effect of the two corticonigral projections per se, but only the behavioral contributions of their target SNr neurons. We have changed the wording to make this clear everywhere in the text.

1.2 While they are mostly careful in how they describe their results, there are a few instances which still imply that they show a behavioral role for the cortical inputs (instead of for the recipient neurons) and need to be changed:

These instances have been modified. Detailed replies below:

1.2.1 Lines 84-85: they should be more precise here, stating that only the recipient neurons are chemogenetically inhibited, not the 'pathways'

This sentence has been modified to clarify that the pathways are only manipulated *ex vivo* (ie., demonstration that SNr firing rate can be impacted in slice), while, indeed, only the recipient neurons are manipulated *in vivo*.

1.2.2 Line 337: 'To probe the functional role of corticonigral transmission without interfering with collateral targets,...' - that is not what they are doing with these experiments – they do not probe the role of the corticonigral transmission, only of the recipient neurons.

We have similarly modified this sentence to clarify that only the M1/M2-recipient neurons in SNr are manipulated *in vivo*.

1.2.3 To make things clear, they should add a clear disclaimer in the discussion that their experiments only speak to the role of the recipient neurons, not of the cortical inputs.

We state throughout the text, as well as in the discussion, that the behavioral experiments reflect manipulation of M1- and M2-recipient SNr-PV+ neurons. We have further clarified this in the discussion with a new paragraph detailing this limitation of our experiments.

1.3 In their discussion, they suggest that the decreased locomotion with inhibition of M1-recipient SNr neurons may be due to a shift away from locomotion and towards fine motor control. However, their results do not seem to support this – they show more head movements (Ext. Fig. 7c), but actually reduced Head&Forepaw movements – which likely would include more fine motor control elements. Their behavioral readouts (in an open field) may not be suited to actually tease this apart, but they should make it clear in their discussion that they don't see indications for a shift towards fine motor control. In addition, they should provide videos of animals when they do not locomote – so that the reader can get an impression how this immobility looks like.

As pointed out, our behavioral readouts from an open field essay are limited. The SNr plays an important role in the fine control of the eyes, tongue, jaw and the rest of the face musculature (Lee et al., 2020; Hikosaka et al., 2020; Rossi et al., 2016). Indeed, such movements cannot be captured with our open field method. Competition between distinct movements could manifest neurally in the brainstem (Usseglio et al., 2020), through lateral connectivity within the SNr, or through basal ganglia-thalamocortical feedback loops (see, for example, Arber & Costa, 2022). We have clarified the wording in the discussion as to not imply that this is what we observe, but rather to suggest possible mechanisms underlying the differences in spontaneous behavior observed in the open field.

We have now provided video examples of both locomotor and stationary episodes, for both M1- and M2-recipient silencing experiments, as well as cut sequences of stationary syllables with and without head movements, extracted through Keypoint-Moseq, as requested (Supplementary Movies 1-6). We have refined the wording of these results to improve clarity (now under Extended Data Fig. 10), as well as the corresponding description of this analysis in the Methods section.

1.4 The behavioral effects of the inhibition of M1-recipient neurons show a clear trend, but are also somewhat variable, with some animals seemingly having the opposite effect (increased displacement and fraction of time mobile; Fig. 6e,f). Could this behavioral variability be due to variability in the injection sites? The authors should provide more information about their injection sites (see also my histology comments). Here, they could potentially show the average or overlaid injections for animals showing a decrease vs. those showing an increase (both for M1 and SNr injections).

We agree with the reviewer that the open field behavior is variable (can be seen clearly in the controls), and one factor may be the injection site. However, there are several others, such as the individual differences between mice. In both M1 and M2 conditions, inter-session changes in control animals were, in fact, more variable than in the experimental groups (Fig. 6g-m). This suggests that there are no systematic issues with the execution of our viral targeting approach. For all animals, the expression of the intersectional construct was verified post-hoc by mCherry expression (Fig. 6b-c). Exclusion criteria have been made clear in the Methods section. Some animals from both M1 and M2 conditions were used for post-hoc electrophysiological verification of DREADD activity *ex vivo*. In these cases (now presented in Extended Data Fig. 9c-d), the cortical injection sites could not be recovered. While not we do not have cortical injection sites for all animals in the behavioral tests, we have now provided a few examples in Extended Data Fig 9b.

2. Histology

2.1 I asked them in the first review to show the injection sites for at the very least all their tracing animals – which they did not do. While they state in their rebuttal that they used the same injection sites for all animals (obviously, but injections can be variable nevertheless), my point is that differences in injection sites in M1 and M2 may lead to different projection patterns in the SNr. Please show the injection sites for all tracing animals (all animals in Fig. 5) and the corresponding pattern in the SNr in the supplement, not just the examples in the main figures.

While we thought the original request to include injection sites for all electrophysiological experiments was unrealistic, this more specific request pertaining only to tracing experiments is indeed feasible. The injection sites and the corresponding density maps for the dual colour axon tracing experiments, as well as injection sites for the dual colour transsynaptic labelling experiments are now presented in Extended Data Fig. 6.

2.2 Fig. 5d: the density plot for the M2-efferents does not seem to resemble the image in Fig. 5c. Again, showing the individual animals may help to explain this discrepancy

The original density plots were 2D dimensional projections of the 3D data to a single plane. To allow a clearer presentation of our data, as requested, we have modified Fig. 5 significantly. We have now split the density plots into 3 regions along the rostrocaudal axis (Fig. 5d). We believe this will allow clearer comparison between the representative section and the summary data and better showcase the topography between the M1- and M2-efferents. We have further added to this data by increasing the number of animals used to 4. All animals are now shown separately in Extended Data Fig. 6, as requested.

2.3 Fig. 5h: they should show an overlay of the density plots to make it easier to see the differential distributions

We have added the requested overlay as spatial differences between the M1-recipient and M2-recipient kernel density estimates, in both coronal and horizontal projections (see updated Fig. 5).

Minor:
- Line 1060: '…was injected in the ipsilateral SNr…' – This gives the impression the injections for the behavior experiments were unilateral – this sentence should be clarified.

This has been clarified to reduce any ambiguity.

- Fig. 6b: They only provide the image for the M1 injections, but the legend says there should also be an image for the M2 injection. They should add that M2 image.

Thank you, this has been corrected.

- Line 1071/1072 – 'left' and 'right' are mixed up here

Thank you, this has been corrected.

- Line 1047: should be ‘mCherry or GFP’

This has also been corrected.

- Please add a note in the text that ‘n’ refers to cells and ‘N’ to animals

We have added such a note to the Methods section and have also explicitly indicated ‘neurons’ or ‘animals’ at every relevant point in the text.

- Line 189: should be ‘characteristic’

This has been corrected, thank you.

- Lines 294-299: add the reference to the figure panels which show the mentioned results

Thank you, we have added a reference to Fig. 5c to this point.

- Line 311: GFP-positive and mCherry-positive?

We have clarified the text to indicate that both GFP-positive and mCherry-positive somata were counted.

Dear Editor,
We have conducted extensive revisions to the manuscript based on the comments of 5 reviewers. These included a new set of behavioral experiments and analysis, tracing experiments, and text revisions.
We have received this last comment by reviewer #5:

Reviewer #5 (Remarks to the Author):
The authors have addressed almost all of my concerns, and the manuscript has clearly improved. I think this is an important contribution and should be published. I have one remaining comment (related to my point 1.4 from the previous round): I am surprised by the strong hesitancy of the authors to show their actual injection sites. While they have now added the injection sites and projection patterns for the tracing (Ex. Fig. 6), they are still not showing the injections for the behavioral animals in Fig. 6. While they don't have the injections sites for all animals (some were used for validation of the silencing), they could have shown the available injection sites and not just the few examples in Ex. Fig. 9. While I don't think this should keep the paper from being published, I am surprised – for a paper which makes a big point about topographic projection patterns, it seems important to actually show the injection sites, since what is actually being hit may strongly affect the outcomes of the experiments. Since they claim that all injection sites have been verified (although they themselves admit that this is not quite correct – they apparently did not verify the cortical injection sites for the animals which were used to verify their DREADD approach – see response to my comment 1.4 from last round), they should also be able to show them. I would strongly recommend for the future to show all injection sites, especially if topography is an important aspect of the studied circuits and outcomes (e.g., behavioral changes) are variable.

Reply:
We acknowledge this reviewer's comment and have provided examples of injections sites for the tracing and the chemogenetic experiments. Unfortunately, we do not have the images for all behavioral experiments despite manual verification. Below is our reply from the previous round:
"...Some animals from both M1 and M2 conditions were used for post-hoc electrophysiological verification of DREADD activity ex vivo. In these cases (now presented in Extended Data Fig. 9c-d), the cortical injection sites could not be recovered. While we do not have cortical injection sites for all animals in the behavioral tests, we have now provided a few examples in Extended Data Fig 9b..."

We appreciate the comment and will strive to do better in future studies.
